# Critical role of VHL/BICD2/STAT1 axis in crystal-associated kidney disease

**DOI:** 10.1038/s41419-023-06185-1

**Published:** 2023-10-13

**Authors:** Wenyan Hao, Hongxian Zhang, Peng Hong, Xin Zhang, Xuyang Zhao, Lulin Ma, Xiaoyan Qiu, Hao Ping, Dan Lu, Yuxin Yin

**Affiliations:** 1 Institute of Systems Biomedicine, Department of Immunology, School of Basic Medical Sciences, NHC Key Laboratory of Medical Immunology, Beijing Key Laboratory of Tumor Systems Biology, Peking University, Beijing, 100191 PR China; 2 Department of Urology, Peking University Third Hospital, Beijing, 100191 PR China; 3 Department of Immunology, School of Basic Medical Sciences, Peking University, Beijing, 100191 PR China; 4 Department of Urology, Beijing Tongren Hospital, Capital Medical University, Beijing, 100730 PR China; 5 Beijing Advanced Innovation Center for Big Data-Based Precision Medicine, Beihang University and Capital Medical University, Beijing Tongren Hospital, Beijing, 100730 PR China

**Keywords:** Cell death and immune response, Ubiquitylation

## Abstract

Nephrolithiasis is highly prevalent and associated with the increased risk of kidney cancer. The tumor suppressor *von Hippel-Lindau* (VHL) is critical for renal cancer development, however, its role in kidney stone disease has not been fully elucidated until now. Here we reported VHL expression was upregulated in renal epithelial cells upon exposure to crystal. Utilizing *Vhl*^*+/mu*^ mouse model, depletion of VHL exacerbated kidney inflammatory injury during nephrolithiasis. Conversely, overexpression of VHL limited crystal-induced lipid peroxidation and ferroptosis in a BICD2-depdendent manner. Mechanistically, VHL interacted with the cargo adaptor BICD2 and promoted its K48-linked poly-ubiquitination, consequently resulting in the proteasomal degradation of BICD2. Through promoting STAT1 nuclear translocation, BICD2 facilitated IFNγ signaling transduction and enhanced IFNγ-mediated suppression of cystine/glutamate antiporter system X_c_^−^, eventually increasing cell sensitivity to ferroptosis. Moreover, we found that the BRAF inhibitor impaired the association of VHL with BICD2 through triggering BICD2 phosphorylation, ultimately causing severe ferroptosis and nephrotoxicity. Collectively, our results uncover the important role of VHL/BICD2/STAT1 axis in crystal kidney injury and provide a potential therapeutic target for treatment and prevention of renal inflammation and drug-induced nephrotoxicity.

## Introduction

Nephrolithiasis or kidney stone disease is one of the most common urological diseases, with an increasing prevalence and incidence worldwide [1, 2]. Most kidney stones consist of calcium oxalate (CaOx) and are recognized as a multifactorial disease [3]. To date, renal function, mineral and lipid metabolism, inflammation, oxidative stress and insulin resistance have been reported to cause CaOx crystal to develop [4, 5]. Reciprocally, CaOx crystal deposits are often accompanied by kidney injury, inflammatory damage as well as tissue remodeling (renal fibrosis) [6]. Therefore, elucidation of mechanism by which nephrolithiasis-mediated inflammation is regulated is critical for prevention of renal fibrosis and also for therapeutic treatment of acute or chronic kidney injury.

In addition to environmental factors, genetic factors are also implicated in the pathophysiology of kidney stone formation [7]. Changes in some candidate genes including *Chloride voltage-gated channel 5 (CLCN5)*, *Calcium sensing receptor (CASR)*, *CoA-disulfide reductase (CDR), Transient receptor potential cation channel subfamily V member 5 (TRPV5)* and *Adenylate cyclase 10 (ADCY10)* have been described to be involved in the pathogenesis of idiopathic hypercalciuria and closely correlated with nephrolithiasis [8]. However, few studies focus on the mechanism by which crystal kidney injury is regulated. The Von Hippel-Lindau (VHL) disease gene was first identified as the tumor suppressor for its genetic disorder in renal cancer development [9]. Utilizing its E3 ubiquitin ligase function, VHL triggers proteasome degradation of hypoxia-inducible factors (HIFs) through promoting its ubiquitination, consequently limiting cellular growth under hypoxia [9]. Additionally, other VHL targets including estrogen receptor α (ERα), Extracellular signal-regulated kinase 5 (ERK5) and Kruppel-like factor 4 (KLF4) have been identified to rationalize the multiple symptoms exhibited in VHL disease [10–12]. Moreover, recent study uncover VHL can act as a negative regulator in host innate immune response through promoting Mitochondrial antiviral-signaling protein (MAVS) instability [13]. However, it is unclear whether, and how, VHL is involved in nephrolithiasis-induced kidney injury and its related inflammatory process.

Several clinical studies showed that treatment with antioxidants, such as vitamin E, vitamin C or zinc could block the progression of tissue injury induced by crystal deposits [14–16], indicating that oxidative stress plays a critical role in the kidney stone diseases. Deregulation of oxidative stress results in lipid peroxidation and ferroptosis, an iron-dependent cell death, which in turn augments inflammatory damage [17]. Notably, recent study shows that activation of interferon-γ (IFNγ) signaling can impair the uptake of cysteine and increase cell susceptibility to ferroptosis through suppression of solute carrier family 7 member 11 (SLC7A11) and solute carrier family 3 member 2 (SLC3A2), two subunits of the glutamate-cystine antiporter system xc- [18]. As one of key cytokines, IFNγ is produced by macrophages, NK cells and effector T cells [19]. However, it remains to be determined whether IFNγ is a primary cause leading to kidney damage or it is a secondary response to the disease. Owing to the similar pathological characteristics to oxidative stress, blockade of IFNγ signaling transduction may provide a promising means against nephrolithiasis-mediated inflammatory damage.

Bicaudal D (BICD) is a dynein activating adaptor protein that plays a key role in organelle and mRNA transport [20]. Mammals possess two BICD orthologues: BICD1 and BICD2 [20]. Both these proteins are built from several coiled-coil domains and serve similar functions, while BICD2 is more abundant in the cell [21]. The two N-terminal CC domain of BICD2 bind to cytoplasmic dynein and dynactin, which have been shown to be important for the transportation from the endoplasmic reticulum to the Golgi compartment [22]. With its third C-terminal coiled coil domain (CC3), BICD2 binds to cargoes such as the small GTPase RAB6 and nucleoporin RANBP2 at the nuclear pores and recruits dynein-dynactin to ensure proper positioning of the nucleus [23]. Recently, another group reported that BICD2 facilitates HIV-1 transportation to the nucleus and enhances viral propagation [24]. However, the role of BICD2 in the transduction of interferon signaling is still elusive.

In this study, we found that VHL expression was upregulated in renal epithelial cells upon exposure to CaOx crystal. Utilizing *Vhl*^*+/mu*^ mouse model, we found that loss of VHL exacerbated kidney stone disease. Mechanistic studies showed that overexpression of VHL enhanced cell resistance to CaOx-induced cell death. Rather than HIFs, we identified BICD2 as the target of VHL, which was essential for the protective effects of VHL on CaOx-induced cell death. BICD2 facilitated IFNγ signaling transduction through promoting STAT1 nuclear translocation and suppressed the expression of SLC3A2 and SLC7A11, consequently enhancing ferroptosis. Reciprocally, VHL promoted BICD2 K48-linked poly-ubiquitination and proteasome-mediated degradation. Additionally, we found the BRAF inhibitor vemurafenib stimulated BICD2 phosphorylation and impaired its association with VHL, consequently increasing the susceptibility to ferroptosis. Our data thus uncover the important role of VHL/BICD2/STAT1 axis in crystal-induced kidney injury and its related inflammatory process.

## Results

### VHL expression is upregulated during nephrolithiasis

Nephrolithiasis is highly related with acute or chronic kidney injury [6]. To study the mechanisms by which the pathological process of nephrolithiasis is modulated, we employed glyoxylate-induced CaOx nephrocalcinosis mouse model. Von Kossa staining of kidney tissue sections and assessment of tissue calcium and iron were performed to confirm the increased CaOx crystal deposition in the kidney (Fig. S1A, B). Additionally, we noticed that increased level of malondialdehyde (MDA) and decreased level of glutathione (GSH) in kidney tissues as well as higher levels of blood urea nitrogen (BUN) and serum creatinine (SCR) in serum were detected from mice treated with glyoxylate (Fig. S1C, D), further supporting the notion that CaOx nephrocalcinosis promotes kidney injury. Through RNA sequencing (RNA-seq) analysis, we found that multiple signaling related with lipid metabolic process, protein transport and ubiquitination were activated during CaOx nephrocalcinosis (Fig. S1E). Among them, we noticed that the mRNA level of *Vhl* was upregulated in kidney following glyoxylate treatment. Ensued RT-qPCR and western blot assays confirm this result (Fig. S2A–C). Through immunohistochemical analysis and semiquantitative evaluation of VHL immunopositivity according to previous studies [25, 26], we found that the expression of VHL was predominantly upregulated in renal epithelial cells (RECs) during CaOx nephrocalcinosis (Fig. S2D, E). To determine whether the upregulation of VHL can be directly induced upon crystal deposition, we co-cultured human normal proximal tubular epithelial cells (HK-2) with calcium oxalate monohydrate (COM) crystal. As expected, the expression of VHL was upregulated in RECs upon crystal stimulation, which was determined by RT-qPCR and western blot assays (Fig. S2F–H).

### Loss of VHL exacerbates nephrolithiasis-induced inflammatory damage

To investigate the role of VHL in progression of nephrolithiasis, we used the *Vhl* mutant mice with heterozygous missense mutation (I117F, conserved with the I151F mutation in human, COSMIC ID: COSM17978) (Fig. S3A–D). Previous report has found that this type of mutation is related with the instability of VHL protein [27]. Accordingly, our data revealed that I151F mutation significantly shortened the half-life of its translated protein as compared with the wild-type *VHL* gene did (Fig. S3E). Similar to the embryonic lethality of the germline knockout of the gene encoding Vhl, the homozygous *Vhl* mutant mice are also lethal, whereas heterozygous *Vhl* mutant (*Vhl*^*+/mu*^) mice are fertile and develop normally. To assess the expression level of Vhl in kidney from *Vhl*^*+/mu*^ mice, we used western blot assay with the anti-VHL antibody. As shown in Fig. S3F, the protein level of Vhl was weakened in the *Vhl*^*+/mu*^ kidney as relative to those in *Vhl*^*+/+*^ kidney. We therefore used the *Vhl*^*+/mu*^ mice to perform the following experiments.

Initially, we treated wild-type or *Vhl*-mutant mice with high (75 mg/kg), medium (60 mg/kg), and low (45 mg/kg) doses of glyoxylate acid (GA), respectively. We observed that a portion of wild-type (14.28%) and *Vhl*^*+/mu*^ mice (50%) died in the treatment of high dose of GA (75 mg/kg). Similar lethal effects were also observed in *Vhl*^*+/mu*^ mice (28.57%) rather than wild-type mice treated with medium dose of GA (60 mg/kg). Both wild-type and *Vhl*^*+/mu*^ mice survived until they were treated with low dose of GA (45 mg/kg). In order to comprehensively analyze the biological role of VHL during nephrolithiasis, we thus committed our ensued experiments with low concentration of GA (45 mg/kg).

**Fig. 1:**
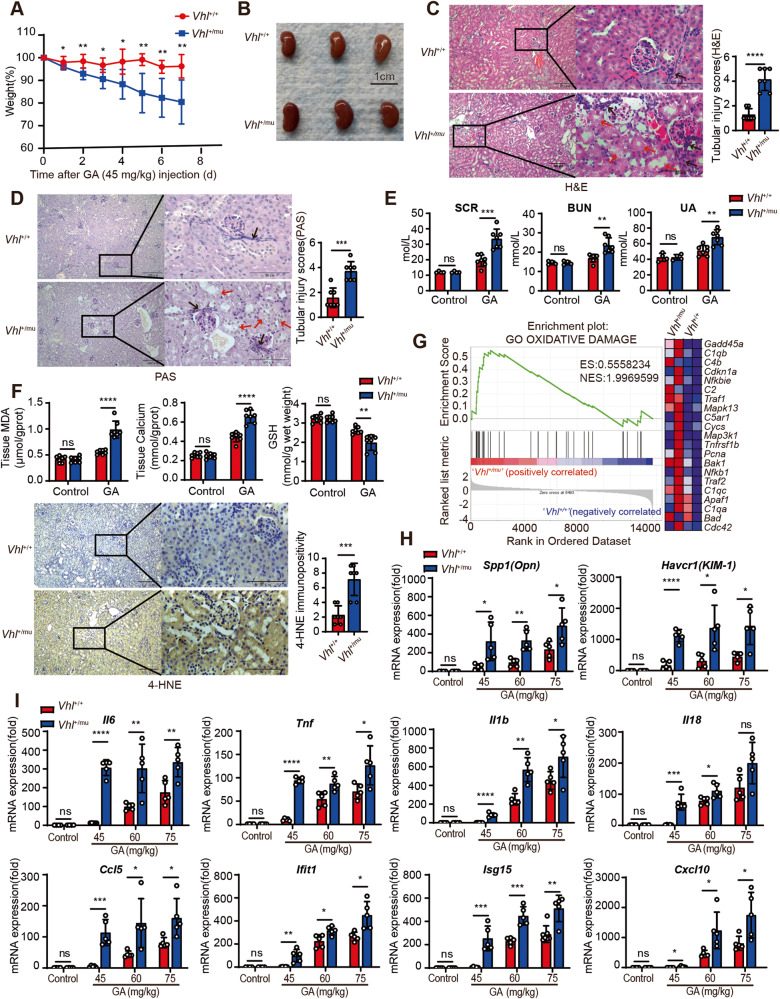
Loss of VHL exacerbates inflammatory damage during nephrolithiasis. **A**–**G** 6-8 weeks old *Vhl*^*+/+*^ and *Vhl*^*+/mu*^ mice were received intraperitoneal injection with either saline (control) or 45 mg/kg of glyoxylate (glyoxylic acid, GA) every day for 7 days and body weights were recorded every day. **A** Body weight changes in *Vhl*^*+/+*^ and *Vhl*^*+/mu*^ mice after intraperitoneal injection with GA (*n* = 7 mice; mean ± SD, *P [1d] = 0.029063, **P [2d] = 0.005223, *P [3d] = 0.012259, *P [4d] = 0.017339, **P [5d] = 0.008039, **P [6d] = 0.002515, **P [7d] = 0.004429). Kidneys were collected, and used for gross examination (**B**), H&E staining (**C**) and PAS staining (**D**). The red arrow indicates injured tubules, the black arrow indicates kidney infiltrating lymphocytes. The kidney damage was scored semiquantitatively in H&E and PAS staining (right) (**C**, **D**). The images are representative of seven mice with similar results. **E** The levels of serum creatinine (SCR), blood urea nitrogen (BUN), blood uric acid (UA) were detected. (control, *n* = 4 mice; GA, *n* = 7 mice; mean ± SD, ns not significant, ***P (SCR) = 0.000267, **P (BUN) = 0.001805, **P (UA) = 0.001902). **F** The levels of malondialdehyde (MDA), Calcium and reduced glutathione (GSH) in kidney tissues were measured (upper panel). Representative images of 4-hydroxynonenal (4-HNE) detection of *Vhl*^*+/+*^ and *Vhl*^*+/mu*^ mice after GA injection and the semiquantitative evaluation of 4-HNE immunopositivity (lower panel). (control, both *Vhl*^*+/+*^and *Vhl*^*+/mu*^, *n* = 7 mice; GA, both *Vhl*^*+/+*^and *Vhl*^*+/mu*^, *n* = 7 mice; mean ± SD, ns not significant; **P = 0.002403, ***P = 0.0003, ****P < 0.0001). **G** The differentially expressed genes between *Vhl*^*+/+*^ and *Vhl*^*+/mu*^ mice after GA injection were analyzed using Gene set enrichment analysis (GSEA) with GO gene sets. ES, enrichment score; NES, normalized enrichment score (*n* = 2 mice). **H**, **I** 6–8 weeks old wild-type mice were received intraperitoneal injection with either saline (control), high (75 mg/kg), medium (60 mg/kg), and low (45 mg/kg) doses of glyoxylate acid (GA) every day, respectively. qRT-PCR analysis was performed to detect the expression levels of genes related with tissue damage (*Spp1* and *Havcr1*) (**H**), inflammatory response (*Il6*, *Tnf, Il1b* and *Il18*) and interferon signaling (*Ccl5, Ifit1, Isg15* and *Cxcl10*) (**I**). (control, *n* = 4 mice; GA, *n* = 5 mice; mean ± SD; ns not significant; *P < 0.05, **P < 0.01, ***P < 0.001 and ****P < 0.0001). Statistical significance was assessed by two-tailed unpaired Student’s t-test. Each data point refers to the number of mice per cohort used per experiment, experiments were repeated at least three times reproducibly, data shown is from one repeat.

Following the glyoxylate treatment, significant weight loss was observed in *Vhl*^*+/mu*^ mice as relative to their wild-type littermate controls (Fig. 1A). Ensued gross tissue evaluation and histological analysis indicated that severe kidney tissue damage was triggered in *Vhl*^*+/mu*^ mice, which was determined by tubular dilation, tubular necrosis, denuded or ruptured tubular basement membranes, epithelial cell apoptosis, intraluminal cast formation, and brush border loss (Fig. 1B, C). Similar results were also detected using PAS staining assay (Fig. 1D). Moreover, the levels of markers of kidney function and tissue damage, including SCR, BUN and UA (uric acid), were significantly increased in serum from *Vhl*^*+/mu*^ mice on day-7 post glyoxylate treatment (Fig. 1E). Reciprocally, the increased levels of MDA, 4-hydroxy-2-nonenal (4-HNE), and calcium as well as the decreased level of glutathione (GSH) were detected in *Vhl*^*+/mu*^ kidney tissues as compared with wild-type kidney tissues during CaOx-induced nephrolithiasis (Fig. 1F). Utilizing the RNAseq assay, we found that the genes related with oxidative damage, IFN signaling as well as IL-6 signaling were enriched in *Vhl*^*+/mu*^ kidneys (Fig. 1G and Fig. S4A, B). We next performed RT-qPCR assay to confirm these results. As shown in (Fig. 1H, I) genes related with tissue damage (*Spp1* and *Havcr1*), inflammatory response (*Il6*, *Tnf, Il1b* and *Il18*) as well as interferon signaling (*Ccl5, Ifit1, Isg15* and *Cxcl10*) were increased in wild-type kidney along with the increase of GA dosage. Conversely, these genes reached the plateau of high level in *Vhl*^*+/mu*^ kidney treated with low dose of GA (Fig. 1H, I), which further support the notion that loss of VHL triggers severe inflammatory damage in kidney during nephrolithiasis.

We next used flow cytometry assay to assess the immune status in kidney following the glyoxylate treatment. As shown in Fig. S4C, greater amounts of CD45^+^ leukocytes were detected in *Vhl*^*+/mu*^ kidneys as compared with *Vhl*^*+/+*^ kidney. Further analysis revealed that the percentages of macrophage (CD11b^+^ F4/80^+^) and T cells (CD3^+^) rather than B cell (CD19^+^) were increased in *Vhl*^*+/mu*^ kidneys during this process (Fig. S4D, E). Notably, the ratio of CD4/CD8 was decreased in *Vhl*^*+/mu*^ kidneys as compared with that in *Vhl*^*+/+*^ kidneys (Fig. S4F), indicating that the enhanced immune response was triggered in *Vhl*^*+/mu*^ kidneys. Above all, our data demonstrate that loss of VHL promotes host inflammatory response and exacerbates nephrolithiasis.

### VHL protects kidney from inflammatory lesion in a HIF -independent manner

To determine whether VHL can directly affect CaOx-induced cell death, we transfected vector encoding VHL into *Vhl*-deficient 786-O cells. Following the treatment of COM, overexpression of VHL remarkably suppressed cell death as compared with control cell did, which was assessed by Annexin V/7-AAD staining, LDH detection and Cell Counting Kit-8 (CCK8) assays (Fig. S5A–C). To assure whether Hypoxia-inducible factor (HIF) signaling is required for the protective effect of VHL or not, we used short hairpin RNA (shRNA) that selectively knocked down the transcription of *HIF1A or HIF2A*, respectively (Fig. S5D). Through analysis by LDH detection and CCK8 assays, we found that loss of HIF1α or HIF2α hardly affected the suppressive effects of VHL on cell death induced by COM treatment (Fig. S5E, F). Our data thus indicate that VHL protects kidney from inflammatory lesion induced by nephrolithiasis in a HIF -independent manner.

### BICD2 is critical for the protective effects of VHL

**Fig. 2:**
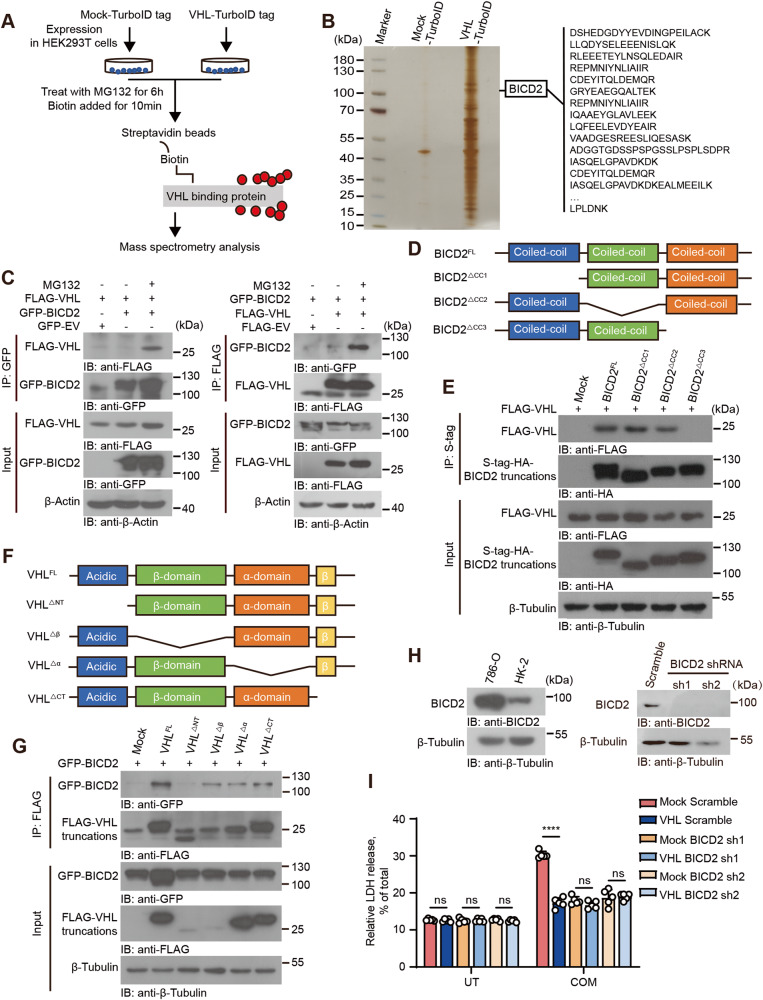
BICD2 is critical for the protective effects of VHL on cell death. **A** Flow chart for VHL-TurboID proximity-based labeling technology. **B** Sliver staining of VHL-associated proteins. BICD2 was identified as a VHL-interacting protein by mass spectrometry, matched peptides corresponding to BICD2 were shown on the right panel. **C** HEK293T cells transfected with the indicated plasmids were treated with or without 10 μM MG132 for 10 h before collection. Cell lysates were immunoprecipitated with appropriate antibodies. The immunoprecipitated proteins were subjected to immunoblot analysis. **D** The schematic of BICD2 truncations. BICD2^FL^, full length BICD2; BICD2^ΔCC1^, BICD2 lacking the first coiled-coil (amino acids 2–269); BICD2^ΔCC2^, BICD2 lacking the second coiled-coil (amino acids 338–537); BICD2^ΔCC3^, BICD2 lacking the third coiled-coil (amino acids 666–824). **E** HEK293T cells were co-transfected with FLAG-tagged-VHL and Mock or plasmids encoding S-HA-tagged BICD2 truncation mutants (above lanes), followed by 10 μM MG132 treatment for 10 h before collection. Cell lysates were immunoprecipitated with S-protein Agarose beads. The immunoprecipitated proteins were subjected to immunoblot analysis with anti-FLAG antibody. **F** The schematic of VHL truncations. VHL^FL^, full length VHL; VHL^ΔNT^, VHL lacking the N-terminal acidic domain (amino acids 1–54); VHL^Δβ^, VHL lacking the beta- domain (amino acids 54–154); VHL^Δα^, VHL lacking the alpha-domain (amino acids 154–192); VHL^ΔCT^, VHL lacking the C-terminus (amino acids 192–213). **G**. HEK293T cells were co-transfected with GFP-tagged-BICD2 and Mock or plasmids encoding FLAG-tagged VHL truncation mutants (above lanes), followed by 10 μM MG132 treatment for 10 h before collection. Cell lysates were immunoprecipitated with anti-FLAG M2 beads and analyzed by immunoblot with anti-GFP antibody. **H** The protein levels of BICD2 in 786-O and HK-2 cells were assessed by immunoblot with anti-BICD2 antibody (left). The effectiveness of BICD2 knockdown in 786-O cells was assessed by immunoblot with anti-BICD2 antibody (right). **I** Endogenous BICD2 was silenced respectively in Mock and VHL stably-expressing 786-O cells. These cells were then treated with 200 μM COM for 24 h. UT untreatment. LDH release was detected (Each data point refers to an individual cell culture within the experiment, *n* = 5 cell cultures, mean ± SD, ns not significant, ****P < 0.0001, two-tailed unpaired Student’s t-test). Experiments were repeated three times reproducibly, data shown is from one repeat.

To investigate the mechanism by which VHL blocks CaOx-induced cell death, we cloned VHL into a mammalian expression vector with a TurboID tag and performed proximity-based labeling screening. Following biotin-affinity capture, the biotinylated proteins were purified and analyzed by mass spectrometry (Fig. 2A). The BICD cargo adaptor 2 (BICD2) was identified as a strong VHL binding partner, which was ensued confirmed by co-immunoprecipitation assay (Fig. 2B, C). Of note, this association between BICD2 and VHL was strengthened in the treatment of the proteasome inhibitor, MG132 (Fig. 2C). We next transfected VHL-FLAG with a series of truncated forms of BICD2 to map the VHL-binding region of BICD2 (Fig. 2D). The results showed that the C-terminal coiled-coil domain of BICD2 is required for its association with VHL (Fig. 2E). Reciprocally, the acidic domain of VHL is necessary for the interaction with BICD2 (Fig. 2F, G).

To determine whether BICD2 is critical for the inhibitory effects of VHL on CaOx-induced cell death, we used shRNA to knockdown endogenous BICD2 (Fig. 2H). We first used the anti-BICD2 antibody and performed the western blot assay to assess the endogenous expression of BICD2 in 786-O cells and HK-2 cells. As shown in Fig. 2H, higher level of BICD2 was detected in 786-O cells as compared with that in HK-2 cells. In order to assess the biological function of BICD2, we thus used shRNA to silence the endogenous BICD2 in 786-O cells expressing high level of BICD2. Through analysis by LDH detection assay, overexpression of VHL elicited little effects on cell death when endogenous BICD2 was silenced following COM treatment (Fig. 2I). Similar result was detected by CCK8 assay (Fig. S5G). Our data thus identify BICD2 as an interactor of VHL that is critical for the inhibitory effects of VHL on CaOx-induced cell death.

### VHL triggers K48-linked poly-ubiquitination of BICD2 and induces its degradation

**Fig. 3:**
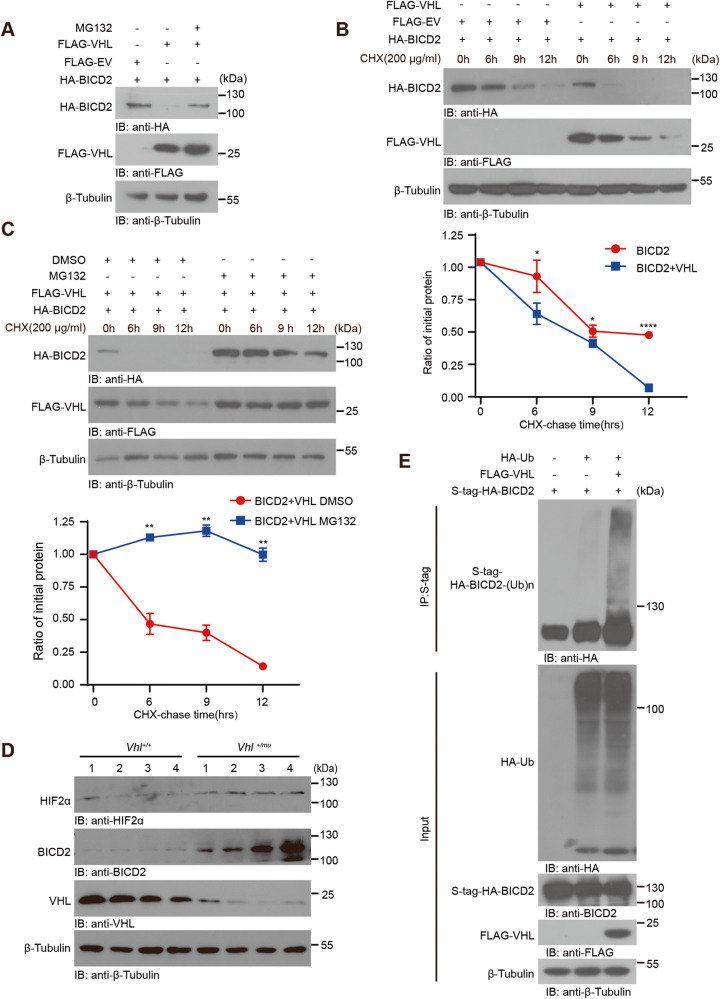
VHL causes BICD2 degradation. **A** HEK293T cells transfected with the indicated plasmids were treated with or without 10 μM MG132 for 10 h before collection. Cell lysates were subjected to immunoblot analysis with appropriate antibodies. **B** Half-life analysis of BICD2 in HEK293T cells. Cells were co-transfected with HA-tagged-BICD2 and FLAG-EV (empty vector) or FLAG-tagged VHL plasmids. Cells were treated with 200 μg/ml cycloheximide (CHX) for indicated times for immunoblot analysis (up). Gray values of BICD2 relative to β-Tubulin were determined by ImageJ software, using for the line chart (down) (*n* = 3 independent experiments, mean ± SD, *P (6 h) = 0.028517, *P (9 h) = 0.040664, ****P (12 h) < 0.0001, two-tailed unpaired Student’s t-test). **C** Half-life analysis of BICD2 in HEK293T cells. Cells were co-transfected with HA-tagged-BICD2 and FLAG-tagged VHL plasmids. Cells treated with 200 μg/ml cycloheximide (CHX) for indicated times with DMSO or 10 μM MG132 were subjected to immunoblot analysis (up). Gray values of BICD2 relative to β-Tubulin were determined by ImageJ software, using for the line chart (down) (*n* = 3 independent experiments, mean ± SD, **P (6 h) = 0.007599, **P (9 h) = 0.004316, **P (12 h) = 0.001876, two-tailed unpaired Student’s t-test). **D** Immunoblot analysis of protein levels of HIF2α, BICD2, VHL in kidneys of *Vhl*^*+/+*^ and *Vhl*^*+/mu*^ mice. β-Tubulin was used for normalization (*n* = 4 mice). **E** In vivo ubiquitination assay of BICD2. HEK293T cells were transfected with indicated plasmids and treated with 10 μM MG132 for 10 h before collection. The whole-cell lysate was subjected to pulldown with S-protein Agarose beads and immunoblot. Statistical significance was assessed by two-tailed unpaired Student’s t-test, *P < 0.05; **P < 0.01; ***P < 0.001; ****P < 0.0001 (**B**, **C**). Experiments were repeated at least three times reproducibly. The immunoblot images shown are from one repeat. The line charts are from 3 independent studies.

As an E3 ubiquitin ligase, VHL can trigger the degradation of a series of protein through inducing k48-linked poly-ubiquitination [9]. To study whether VHL can also cause BICD2 degradation, we co-transfected vectors encoding VHL and BICD2 into HEK293T cells with or without MG132 treatment. Through analysis by western blot assay, overexpression of VHL restricted BICD2 expression, which were rescued by the treatment of MG132 (Fig. 3A). Moreover, we used the protein synthesis inhibitor, cycloheximide (CHX), to assess the half-life of protein. As shown in Fig. 3B, overexpression of VHL accelerated the degradation of BICD2 as compared with empty vector did. Conversely, the stimulatory effects of VHL on BICD2 degradation can be blocked by the treatment of MG132 (Fig. 3C). Consistent with these results in vitro, we also assessed the status of BICD2 in *Vhl*^*+/+*^ and *Vhl*^*+/mu*^ kidney and found that the expression of BICD2 was upregulated in *Vhl*^*+/mu*^ kidney as compared with *Vhl*^*+/+*^ (Fig. 3D). Our data thus demonstrate that VHL causes BICD2 degradation.

To study whether VHL triggers BICD2 ubiquitination, we performed the co-immunoprecipitation assay and found enhanced ubiquitination of BICD2 in presence of VHL (Fig. 3E). To determine which kind of ubiquitin modification was triggered by VHL, we used a series of mutants of HA-tagged ubiquitin in which remained only one of the seven lysine sites (K6, K11, K27, K29, K33, K48 and K63) or just the N-terminal methionine (K0). Through the co-immunoprecipitation assay, we found that VHL selectively induced the K48-linked poly-ubiquitination of BICD2 (Fig. S6A). Collectively, our data demonstrate that VHL induces BICD2 degradation through promoting K48-linked poly-ubiquitination of BICD2.

### BICD2 enhances COM crystal-induced cell death

**Fig. 4:**
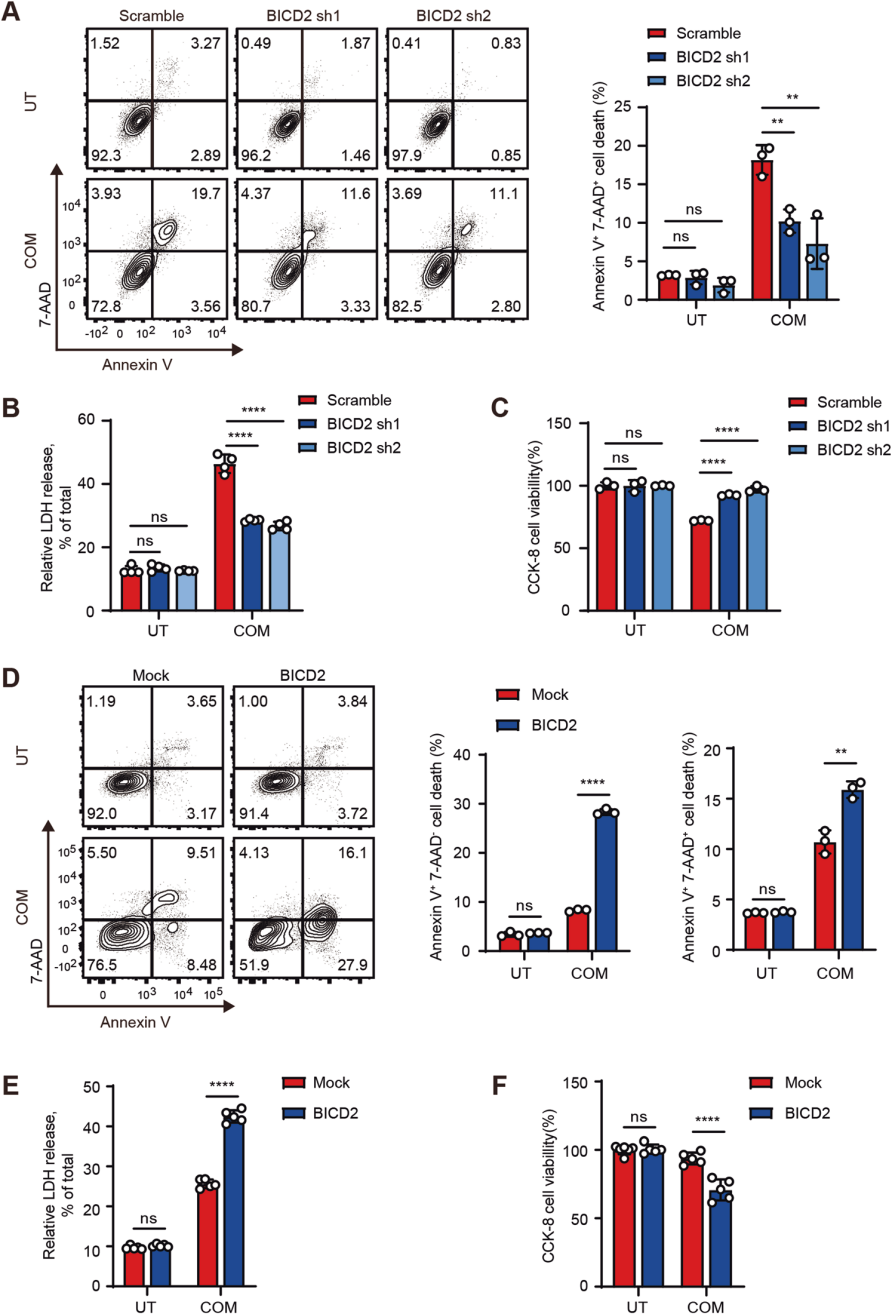
BICD2 enhances COM crystal-induced cell death. **A**–**C** BICD2-deficient or control 786-O cells were treated with 200 μM COM for 24 h. UT untreatment. **A** The death cells were assessed by flow cytometric analysis of Annexin V/7-AAD staining. The percentages of Annexin V^+^ 7-AAD^+^ cells were analyzed (right) (*n* = 3 cell cultures, mean ± SD, ns not significant, **P = 0.004969, **P = 0.007848). **B** Lactate dehydrogenase (LDH) release was detected (*n* = 4 cell cultures, mean ± SD, ns not significant, ****P < 0.0001). **C** The cell viability was detected by Cell counting Kit-8 (CCK-8) (*n* = 3 cell cultures, mean ± SD, ns not significant, ****P < 0.0001). **D–F** Mock or BICD2 stably-expressing HK-2 cells were treated with 200 μM COM for 24 h. UT untreatment. **D** The death cells were assessed by flow cytometric analysis of Annexin V/7-AAD staining. The percentages of Annexin V^+^ 7-AAD^-^ cells and Annexin V^+^ 7-AAD^+^ cells were analyzed separately (*n* = 3 cell cultures, mean ± SD, ns not significant, ****P < 0.0001; **P = 0.003090). **E** LDH release was detected (*n* = 5 cell cultures, mean ± SD, ns not significant, ****P < 0.0001). **F** The cell viability was detected by Cell counting Kit-8 (CCK-8) (*n* = 5-6 cell cultures, mean ± SD, ns not significant, ****P < 0.0001). Statistical significance was assessed by two-tailed unpaired Student’s t-test, *P < 0.05; **P < 0.01; ***P < 0.001; ****P < 0.0001. Each data point refers to an individual cell culture within the experiment. Experiments were repeated at least three times reproducibly, data shown is from one repeat.

Since loss of BICD2 neutralizes the suppressive effect of VHL on cell death, we thus focused on the role of BICD2 in modulation of CaOx-induced cell death. As shown in Fig. 4A, fewer Annexin V^+^ 7-AAD^+^ cells were detected when endogenous BICD2 was silenced as compared with control cells. Similar results were also detected by LDH detection and CCK8 assays (Fig. 4B, C). Accordingly, we also transfected the vector encoding BICD2 into HK-2 cells and then treated with COM. As shown in Fig. 4D–F, the percentage of dying cells was increased when BICD2 was transfected into HK-2 cells as compared with control cells. Our data thus demonstrate that BICD2 enhances COM crystal-induced cell death.

### BICD2 increases cell sensitivity to ferroptosis

In addition to LDH, we found that greater amount of ATP was released from BICD2-expressing cells when cells were treated with COM (Fig. S7A). Reciprocally, loss of BICD2 decreased ATP leakage as relative to control cells (Fig. S7B). Additionally, we noticed that loss of BICD2 limited intracellular Reactive oxygen species (ROS) production, while higher level of ROS was detected in BICD2-expressing cells upon exposure to COM (Fig. S7C, D), indicating that BICD2 may augment oxidative stress induced by COM treatment. To further confirm our hypothesis, we used hydrogen peroxide (H_2_O_2_) to treat cells in presence or absence of BICD2. As shown in Fig. S7E, F, loss of BICD2 blocked H_2_O_2_-induced cell death and limited ATP leakage. Conversely, greater amounts of LDH and ATP were released into extracellular matrix when BICD2 was transfected into HK-2 cells following the treatment of H_2_O_2_ (Fig. S7G, H). Our data thus demonstrate that enforced expression of BICD2 enhances CaOx-induced oxidative stress.

**Fig. 5:**
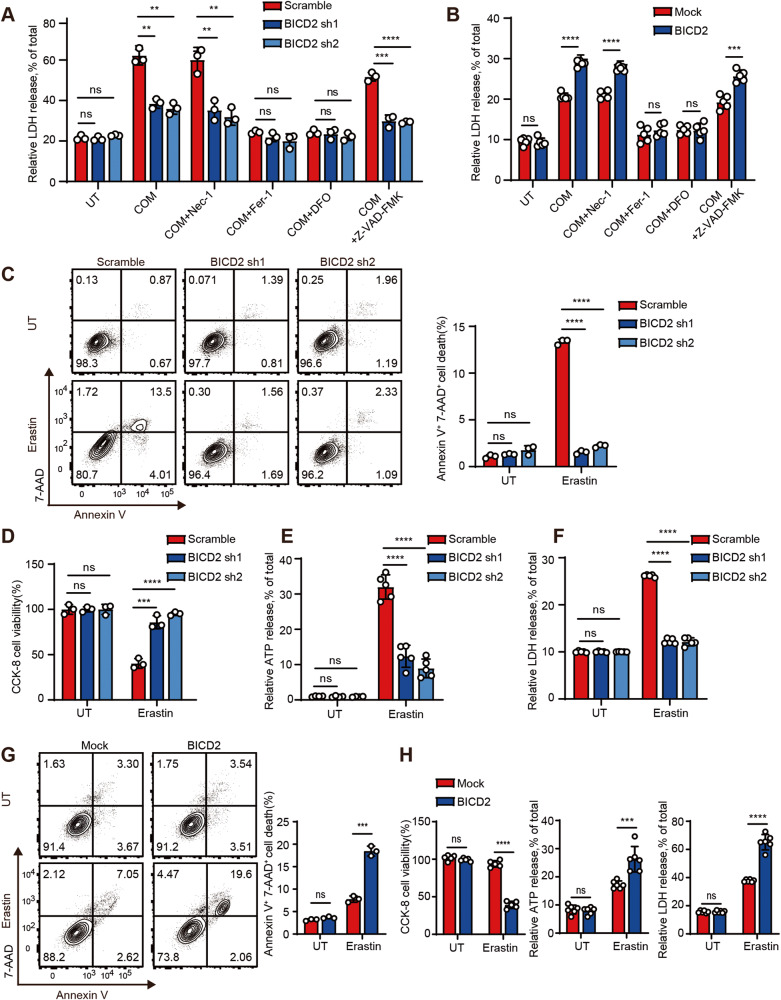
BICD2 increases cell sensitivity to ferroptosis. BICD2-deficient or control 786-O cells (**A**), Mock or BICD2 stably-expressing HK-2 cells (**B**) were treated with 200 μM COM and a series of cell death inhibitors for 24 h. The supernatants were collected and used to LDH release assay. (UT untreatment; Nec-1, 2 μM necrostatin-1; Fer-1, 5 μM ferrostatin-1; DFO, 10 μM deferoxamine; 20 μM Z-VAD-FMK). **A** Assessment of LDH release from BICD2-deficient or control 786-O cells (*n* = 3 cell cultures, mean ± SD, ns not significant, **P = 0.001468, **P = 0.001171 (COM); **P = 0.005380, **P = 0.002815 (COM + Nec-1); ***P = 0.000476, ****P < 0.0001 (COM + Z-VAD-Fmk)). **B** Assessment of LDH release from Mock or BICD2 stably-expressing HK-2 cells (*n* = 5 cell cultures, mean ± SD, ns not significant, ****P < 0.0001, ***P = 0.000208). **C**–**F** BICD2-deficient or control 786-O cells were treated with 1 μM ferroptosis agonist Erastin for 18 h. UT untreatment. **C** Flow cytometric analysis of dying cells using Annexin V/7-AAD staining. The percentages of Annexin V^+^ 7-AAD^+^ cells were analyzed (right) (*n* = 3 cell cultures, mean ± SD, ns not significant, ****P < 0.0001). **D** The CCK-8-based cell viability of BICD2-deficient or control 786-O cells (*n* = 3 cell cultures, mean ± SD, ns not significant, ***P = 0.000932, ****P < 0.0001). **E** ATP release assay of BICD2-deficient or control 786-O cells (*n* = 5 cell cultures, mean ± SD, ns not significant, ****P < 0.0001). **F** Assessment of LDH release from BICD2-deficient or control 786-O cells (*n* = 5 cell cultures, mean ± SD, ns not significant, ****P < 0.0001). **G**, **H** Mock or BICD2 stably-expressing HK-2 cells were treated with 1.5 μM ferroptosis agonist Erastin for 18 h. UT untreatment. **G** Flow cytometric analysis of dying cells using Annexin V/7-AAD staining. The percentages of Annexin V^+^ 7-AAD^+^ cells were analyzed (right) (*n* = 3 cell cultures, mean ± SD, ns not significant, ***P = 0.000213). **H** The CCK-8-based cell viability, the ATP and LDH release assays of Mock or BICD2 stably-expressing HK-2 cells (*n* = 5–6 cell cultures, mean ± SD, ns not significant, ***P = 0.000840, ****P < 0.0001). Statistical significance was assessed by two-tailed unpaired Student’s t-test, **P < 0.01; ***P < 0.001; ****P < 0.0001. Each data point refers to an individual cell culture within the experiment. Experiments were repeated at least three times reproducibly, data shown is from one repeat.

We next employed a series of cell death inhibitors to treat BICD2-deficient or control cells during COM treatment. As shown in Fig. 5A, compared with the reduced amount of LDH release from BICD2-deficient cells in the treatment of other cell death inhibitors, the amount of LDH was similar between BICD2-deficient cells and control cell in the treatment of the ferroptosis inhibitor ferrostatin-1 (Fer-1) or iron chelation deferoxamine (DFO). Furthermore, both Fer-1 and DFO can revert the enhanced cell death induced by BICD2 (Fig. 5B), suggesting that BICD2 predominantly triggers ferroptosis upon exposure to COM. To further confirm our hypothesis, we used ferroptosis agonist Erastin to treat BICD2-deficient or control cells. As shown in Fig. 5C, fewer percentage of Annexin V^+^ 7-AAD^+^ dying cells was detected when endogenous BICD2 was silenced following Erastin treatment. Similar results were also observed by CCK8, ATP assessment and LDH detection assays (Fig. 5D–F). Reciprocally, overexpression of BICD2 increased cell sensitivity to Erastin treatment (Fig. 5G, H). In addition to Erastin treatment, we also used RLS3, another type of Ferroptosis agonist. As shown in Fig. S8A–F, loss of BICD2 increased cell resistance to RSL3, while overexpression of BICD2 enhanced RSL3-induced ferroptotic cell death. Taken together, our data demonstrate that BICD2 augments oxidative stress and increases cell sensitivity to ferroptosis.

### Vemurafenib stabilizes BICD2 through interrupting its association with VHL

**Fig. 6:**
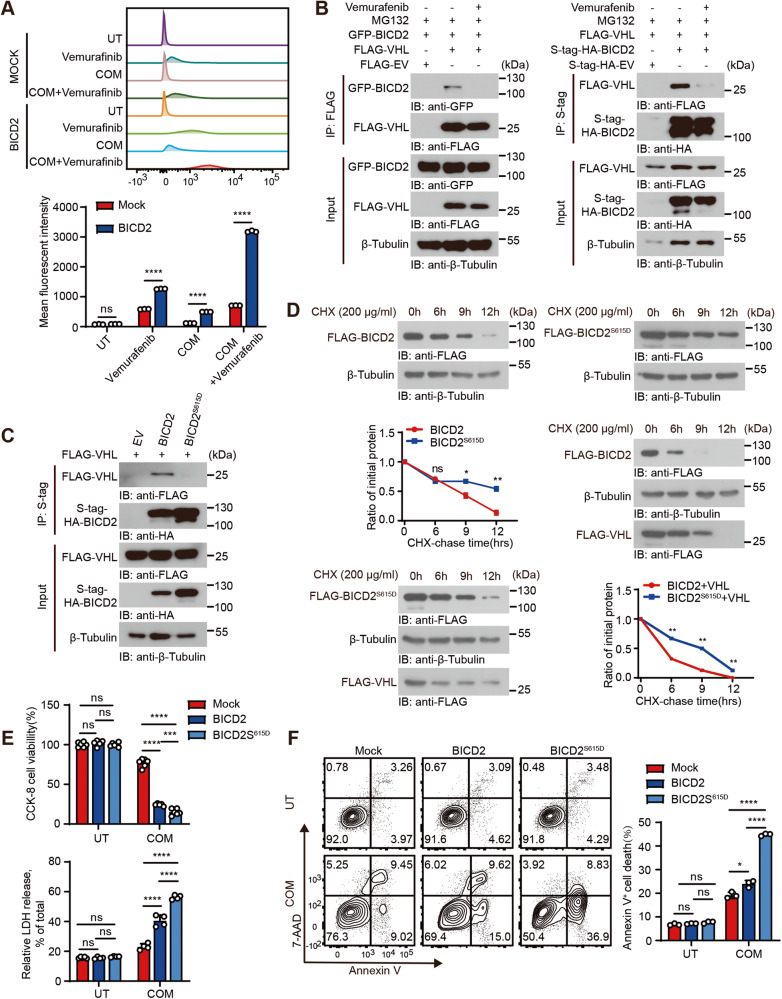
Vemurafenib triggers BICD2 phosphorylation and interrupts its association with VHL. **A** Mock or BICD2 stably-expressing HK-2 cells were treated with 10 μM Vemurafenib, 200 μM COM, 10 μM Vemurafenib and 200 μM COM for 2 h, respectively. UT untreatment. Cells were collected and used to measure the intracellular ROS production (*n* = 3 cell cultures, mean ± SD, ns not significant, ****P < 0.0001). **B** HEK293T cells transfected with the indicated plasmids were treated with or without 10 μM Vemurafenib for 12 h before collection. Cell lysates were immunoprecipitated with appropriate antibodies, ensued analyzed by immunoblot assay. **C** HEK293T cells were transfected with the indicated plasmids, followed by 10 μM MG132 treatment for 10 h before collection. Cell lysates were immunoprecipitated with S-protein Agarose beads and analyzed by immunoblot with anti-FLAG antibody. **D** Half-life analysis of BICD2 and mutant BICD2 ^S615D^ with VHL or not in HEK293T cells. Cells were transfected with the indicated plasmids. Cells treated with 200 μg/ml cycloheximide (CHX) for indicated times were subjected to immunoblot analysis. Gray values of BICD2 and BICD2 ^S615D^ relative to β-Tubulin were determined by ImageJ software, using for the line chart (mean ± SD, ns not significant; *P (9 h) = 0.016441, **P (12 h) = 0.008480 (left panel); **P (6 h) = 0.003575, **P (9 h) = 0.001232, **P (12 h) = 0.004382 (right panel)). The immunoblot images shown are from one repeat. The line charts are from 3 independent studies. **E**, **F** Mock, BICD2, BICD2 ^S615D^ stably-expressing HK-2 cells were treated with 200 μM COM for 24 h. UT untreatment. **E** The CCK-8-based cell viability and LDH release were detected. (*n* = 4–6 cell cultures, mean ± SD, ns not significant, ***P = 0.000821, ****P < 0.0001). **F** The death cells were assessed by flow cytometric analysis of Annexin V/7-AAD staining. The percentages of Annexin V^+^ cells were analyzed (*n* = 3 cell cultures, mean ± SD, ns not significant, *P = 0.013543, ****P < 0.0001). Statistical significance was assessed by two-tailed unpaired Student’s t-test, *P < 0.05; **P < 0.01; ***P < 0.001; ****P < 0.0001. Each data point refers to an individual cell culture within the experiment. Experiments were repeated at least three times reproducibly, data shown is from one repeat.

It has been reported that vemurafenib can trigger phosphorylation on the serine residue S615 of BICD2 [28]. Moreover, supplementation of vemurafenib elicited the stimulatory effects on cell sensitivity to ferroptosis [29]. We thus hypothesized that BICD2 phosphorylation might contribute to the positive effects of vemurafenib on ferroptosis. To this end, we treated BICD2-expressing or control cells with vemurafenib and/or COM. Through DC-FDA staining assay, increased level of Reactive Oxygen Species (ROS) was detected in both BICD2-expressing or control cells treated with vemurafenib. Moreover, overexpression of BICD2 significantly enhanced the upregulation of ROS production in the treatment of vemurafenib and/or COM. Of note, highest level of lipid peroxidation was detected in BICD2-expressing cells treated with vemurafenib plus COM (Fig. 6A). Our data thus indicate that BICD2 is implicated in vemurafenib-induced lipid peroxidation.

Through co-immunoprecipitation assay, we found that supplementation of vemurafenib impaired the association of BICD2 with VHL (Fig. 6B). To mimic the phosphorylation of BICD2 by Vemurafenib, we mutated S to D on the serine residue S615 of BICD2. Consistent with the vemurafenib results, mutant BICD2 (S615D) hardly interacted with VHL as compared with the association of VHL with wild-type BICD2 (Fig. 6C). Moreover, overexpression of VHL exerted little effects on the ubiquitin modification of mutant BICD2 (S615D) (Fig. S9A). To determine whether BICD2 phosphorylation affects its protein stability, we transfected vectors encoding wild-type or mutant BICD2^S615D^ into 293T cells with or without VHL. Following the CHX treatment, ectopic expression of VHL significantly accelerated the degradation of BICD2 as compared with control cells. Moreover, S615D mutant prolonged the half-life of BICD2 and elicited resistance to VHL-mediated BICD2 degradation (Fig. 6D). Upon exposure to COM crystal, increased percentage of dead cells was detected when mutant BICD2^S615D^ was transfected as compared with control cells or wild-type BICD2-expressing cells (Fig. 6E, F). Our data thus indicate that BICD2 phosphorylation by vemurafenib impairs its association with VHL and enhances BICD2 protein stability, eventually increasing cell sensitivity to ferroptosis.

### BICD2 interacts with transcription factor STAT1

**Fig. 7:**
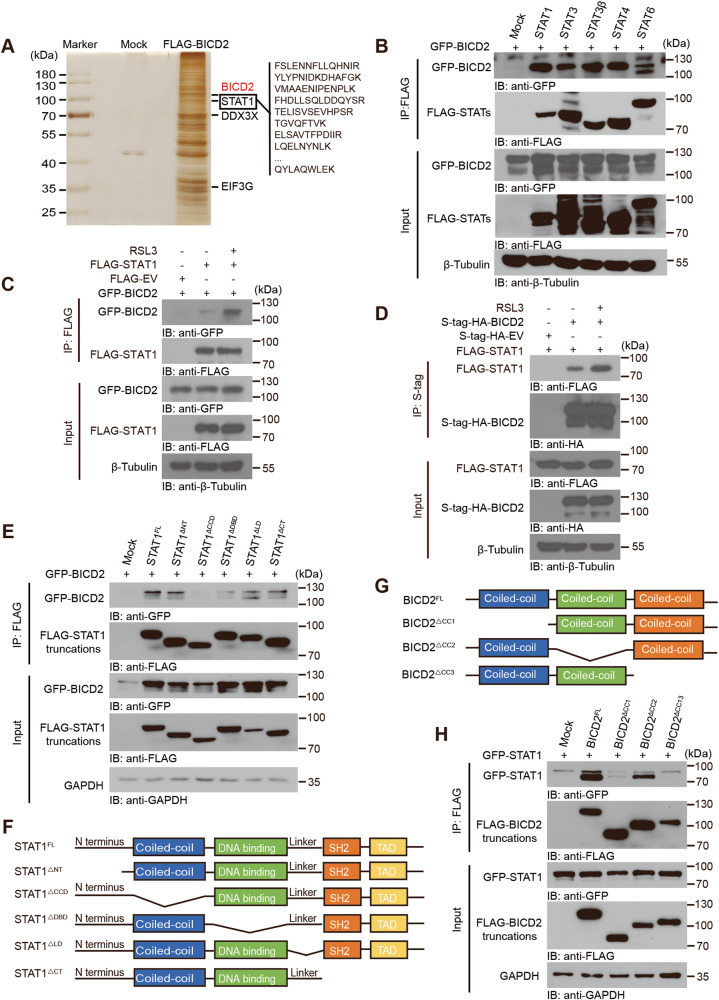
BICD2 interacts with the transcription factor STAT1. **A** Mass spectrum analysis of BICD2-associated proteins. Mock or FLAG-tagged-BICD2 plasmid was transfected into HEK293T cells, and FLAG-tagged proteins were enriched by anti-FLAG M2 beads. Proteins that interact with BICD2 are indicated on the right (outlined text). **B** HEK293T cells were co-transfected with GFP-tagged-BICD2 and Mock or FLAG-tagged STAT1, STAT3, STAT3β, STAT4 or STAT6 plasmids (above lanes). Cell lysates were immunoprecipitated with anti-FLAG antibody and analyzed by immunoblot with anti-GFP antibody. HEK293T cells were transfected to express BICD2 and Mock or STAT1, with or without 1 μM RSL3 treatment for 6 h before collection. Cell lysates were immunoprecipitated with anti-FLAG antibody (**C**) or S-protein Agarose beads (**D**), followed by immunoblot analysis with anti-GFP antibody (**C**) or anti-FLAG antibody (**D**). **E** HEK293T cells were co-transfected with GFP-tagged-BICD2 and Mock or plasmids encoding FLAG-tagged STAT1 truncation mutants (above lanes). Cell lysates were immunoprecipitated with anti-FLAG antibody and analyzed by immunoblot with anti-GFP antibody. **F** The schematic of STAT1 truncations. STAT1^FL^, full length STAT1; STAT1^ΔNT^, STAT1 lacking the N terminus (amino acids 1–130); STAT1^ΔCCD^, STAT1 lacking the coiled-coil domain (amino acids 130–315), STAT1^ΔDBD^, STAT1 lacking the DNA-binding domain (amino acids 315–488).; STAT1^ΔLD^, STAT1 lacking the linker domain (amino acids 488–576); STAT1^ΔCT^, STAT1 lacking the C terminus (amino acids 576–750). **G** The schematic of BICD2 truncations. BICD2^FL^, full length BICD2; BICD2^ΔCC1^, BICD2 lacking the first coiled-coil (amino acids 2–269); BICD2^ΔCC2^, BICD2 lacking the second coiled-coil (amino acids 338–537), BICD2^ΔCC3^, BICD2 lacking the third coiled-coil (amino acids 666–824). **H** HEK293T cells were co-transfected with GFP-tagged-STAT1 and Mock or plasmids encoding FLAG-tagged BICD2 truncation mutants (above lanes). Cell lysates were immunoprecipitated with anti-FLAG antibody and analyzed by immunoblot with anti-GFP antibody. Experiments were repeated three times reproducibly. The immunoblot images shown are from one repeat.

To investigate the molecular mechanism by which BICD2 positively modulates ferroptosis, we used immunoprecipitation followed by mass spectrometry (MS) to identify BICD2-associated proteins. As shown in Fig. 7A, multiple reported BICD2-associated proteins, including DDX3X and EIF3G, were identified. However, a notable finding was the identification of STAT1 as the BICD2-binding protein. Subsequent co-immunoprecipitation assay further confirmed the physical association between BICD2 and STAT1 (Fig. 7B). We also noticed that all other STAT proteins interacted, to varying extents, with BICD2, but STAT1 had the greatest interaction (Fig. 7B). Interestingly, we found that the association of BICD2 with STAT1 were enhanced in presence of RSL3 or COM as compared with cells under quiescent condition (Fig. 7C, D and Fig. S9B, C). We next co-expressed BICD2-GFP with a series of truncated forms of STAT1 to map the BICD2-binding region of STAT1. As shown in Fig. 7E, F, the coiled-coil domain of STAT1 was essential for its interaction with BICD2. Reciprocally, both the N-terminal and C-terminal coiled-coil domain of BICD2 were indispensable for its association with STAT1 as determined by co-immunoprecipitation assay (Fig. 7G, H). Our findings thus observe the interaction between BICD2 and STAT1.

### BICD2 facilitates STAT1 nuclear translocation upon IFNγ and suppresses the expression of SLC7A11 and SLC3A2

**Fig. 8:**
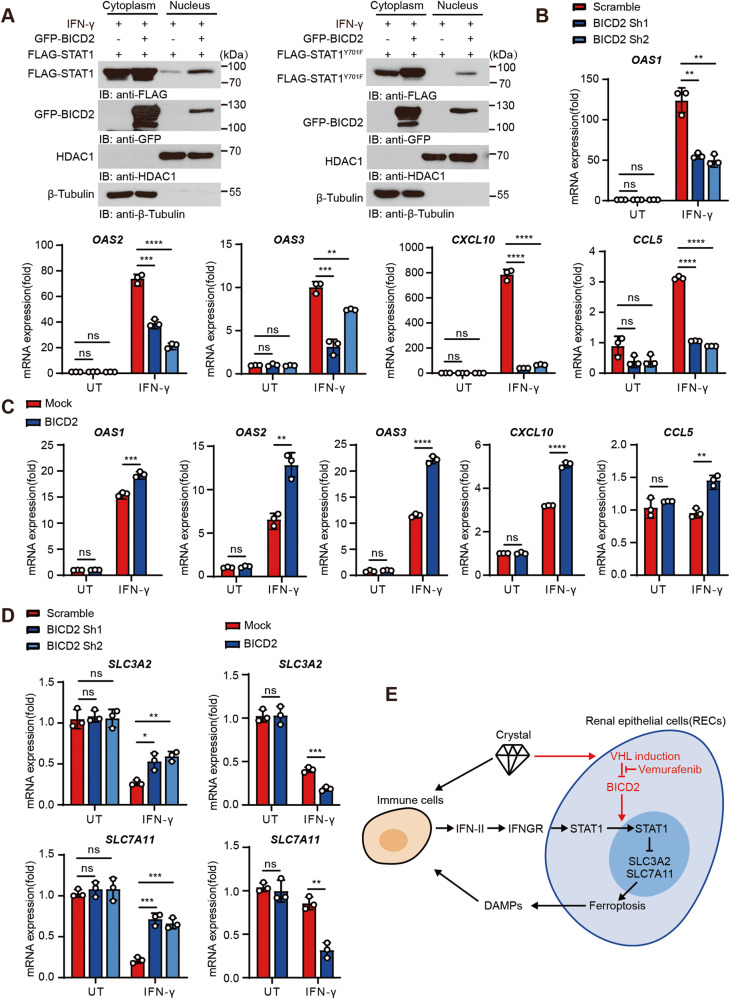
BICD2 facilitates STAT1 nuclear translocation upon IFNγ stimulation. **A** HEK293T cells transfected with the indicated plasmids were treated with 20 nM IFN-γ for 2 h before collection. Nuclear and cytoplasmic extracts were isolated and analyzed by immunoblot assay. **B** BICD2-deficient or control 786-O cells were treated with 20 nM IFN-γ. UT untreatment. qRT-PCR analysis was performed to detect the expression levels of the transcription of interferon-stimulated genes (ISGs), including *OAS1, OAS2, OAS3, CXCL10* and *CCL5* (*n* = 3 cell cultures, mean ± SD, ns not significant; **P = 0.001811, **P = 0.001726 (*OAS1*); ***P = 0.000170 (*OAS2*); ***P = 0.000370, **P = 0.002315 (*OAS3*); ****P < 0.0001). **C** Mock or BICD2 stably-expressing HK-2 cells were treated with 20 nM IFN-γ. UT untreatment. qRT-PCR analysis was performed to detect the expression levels of the transcription of ISGs, including *OAS1, OAS2, OAS3, CXCL10* and *CCL5* (*n* = 3 cell cultures, mean ± SD, ns not significant; ***P = 0.000488 (*OAS1*); **P = 0.002410 (*OAS2*); **P = 0.001121 (*CCL5*); ****P < 0.0001). **D** BICD2-deficient or control 786-O cells and Mock or BICD2 stably-expressing HK-2 cells were treated with 20 nM IFN-γ. UT untreatment. qRT-PCR assay was performed to detect the expression levels of *SLC3A2* and *SLC7A11* (*n* = 3 cell cultures, mean ± SD, ns not significant; *P = 0.012951, **P = 0.001104, ***P = 0.000554 (*SLC3A2*); ***P = 0.000454, ***P = 0.000476, **P = 0.001189 (*SLC7A11*)). **E** Graphical Abstract. VHL expression is induced in renal epithelial cells upon exposure to CaOx crystal. Through triggering BICD2 K48-linked poly-ubiquitination and degradation, VHL blocks BICD2-mediated STAT1 nuclear translocation and maintains the expression of SLC3A2 and SLC7A11, eventually enhancing cell resistance to lipid peroxidation. Moreover, the BRAF inhibitor Vemurafenib can stimulate BICD2 phosphorylation and impair its association with VHL, consequently increasing the susceptibility to ferroptosis. Statistical significance was assessed by two-tailed unpaired Student’s t-test, *P < 0.05; **P < 0.01; ***P < 0.001; ****P < 0.0001 (**B–D**). Each data point refers to an individual cell culture within the experiment. Experiments were repeated at least three times reproducibly, data shown is from one repeat (**A–D**).

By definition, the dynein adaptor BICD2 engages both cargo and dynein and promotes intracellular transport. To determine whether BICD2 affects the subcellular localization of STAT1, we employed the nuclear-cytoplasmic fractionation assay. As shown in Fig. S9D, overexpression of BICD2 elicited little effects on nuclear translocation of STAT1 under quiescent condition. Additionally, we also used the vector encoding tyrosine-mutated (Y701F) STAT1, which interrupts the nuclear accumulation of STAT1 following the engagement of IFN. Similar to wild-type STAT1, the subcellular localization of mutant STAT1 were predominantly in the cytoplasm in presence or absence of BICD2 (Fig. S9E). Since STAT1 is an essential transcription factor of IFN signaling, we then employed type II IFN to stimulate BICD2-expressing or control cells. As shown in Fig. 8A left, overexpression of BICD2 promoted the nuclear translocation of STAT1 following IFNγ stimulation. More importantly, BICD2 triggered the nuclear accumulation of tyrosine-mutated (Y701F) STAT1 following IFNγ stimulation (Fig. 8A right). To determine whether BICD2 facilitates IFN signaling transduction, we used RT-qPCR to detect the transcription of interferon-stimulated genes (ISGs). Compared with control cells, loss of BICD2 limited the transcription of ISGs including *OAS1, OAS2, OAS3, CXCL10* and *CCL5* (Fig. 8B). Reciprocally, overexpression of BICD2 enhanced STAT1-mediated ISGs transcription (Fig. 8C).

In addition to transactivation of ISGs, activation of IFN signaling suppresses the expression of SLC3A2 and SLC7A11, two subunits of the glutamate-cystine antiporter system xc-, impairs the uptake of cystine, consequently promoting cell lipid peroxidation and ferroptosis [18]. We thus hypothesize that BICD2 may enhance IFNγ-mediated suppression of SLC3A2 and SLC7A11. To this end, we used IFNγ to stimulate BICD2-deficient or control cells. As shown in Fig. 8D left, higher transcriptional levels of SLC3A2 and SLC7A11 were detected in BICD2-deficent cells as compared with control cells following IFNγ stimulation. Accordingly, overexpression of BICD2 enhanced the downregulation of SLC3A2 and SLC7A11 induced by IFNγ stimulation (Fig. 8D right). Our data thus demonstrate that the cargo adaptor BICD2 facilitates STAT1 nuclear translocation and enhances IFNγ-mediated suppression of SLC3A2 and SLC7A11, eventually promoting ferroptotic cell death.

Collectively, our data demonstrate that VHL expression is upregulated in renal epithelial cells upon exposure to CaOx crystal. Through triggering BICD2 K48-linked poly-ubiquitination and degradation, VHL blocks BICD2-mediated STAT1 nuclear translocation and maintains the expression of SLC3A2 and SLC7A11, eventually enhancing cell resistance to lipid peroxidation. Moreover, our data uncover the BRAF inhibitor vemurafenib can stimulate BICD2 phosphorylation and impair its association with VHL, consequently increasing the susceptibility to ferroptosis (Fig. 8E).

## Discussion

Kidney stone disease is caused by factors such as mineral and lipid metabolism, inflammation or oxidative stress and is characterized by impaired kidney-filtration function [5]. The damage of the renal epithelial cells triggers acute inflammatory lesion and increases morbidity and mortality [30]. Unfortunately, kidney stones-related inflammation still remains a headache condition which lacks specific tools for treatment until now. Emerging evidence supports the concept that ferroptosis, among all the types of cell death, plays critical role in the pathophysiology of acute or chronic kidney injury [31], highlighting ferroptosis as a promising target for treatment of kidney stones-related inflammation.

In addition to inflammation, chronic kidney stone diseases have an overall risk ratio of 1.76 (95% CI 1.24–2.49) for renal carcinoma [32]. As the tumor suppressor, *VHL* is highly frequently mutated in renal carcinomas [9]. Although it has been well-documented that VHL triggers HIFs instability and inhibits tumor growth, its role in the kidney cancer development has not been fully elucidated until now. In this study, we show that the expression of VHL is upregulated in renal epithelial cells upon exposure to CaOx crystals. Utilizing kidney stone forming mice model, *Vhl*^*+/mu*^ mice exhibited severe inflammatory damage during nephrolithiasis. Further analysis reveals that VHL attenuates CaOx-induced oxidative stress and limits ferroptosis. Interestingly, it has been reported that presence of HIF1α also blocks ferroptotic cell death, which contradicts with our data. Using shRNA to specific knockdown endogenous HIFs or BICD2, we find that BICD2 rather than HIFs is required for the protective effects of VHL on CaOx-induced cell death. In light of the stimulatory role of VHL deficiency on crystal-induced inflammation and tissue remodeling, our results thus provide a bridge linking the two carcinogenic factors and offer new strategies for the prevention of kidney cancer.

IFNγ is implicated in multiple inflammatory diseases [19]. Through engagement with IFNγ receptors, IFNγ activates JAK/STAT1 signaling and stimulates transcription of ISGs [19]. Notably, recent study shows that activation of IFNγ signaling can impair the uptake of cysteine and increase cell susceptibility to ferroptosis through suppression of SLC7A11 and SLC3A2 [18]. In light of the key role of STAT1 in IFNγ signaling, it is conceivable that nuclear accumulation of STAT1 may contribute to this process. Here, we find that BICD2 acts as the cargo adaptor of STAT1 that facilitated its nuclear translocation. Accordingly, overexpression of BICD2 promoted IFNγ signaling transduction as determined by the upregulation of ISGs. Moreover, BICD2 strengthened the IFNγ-mediated suppression of SLC3A2 and SLC7A11. Considering the stimulatory effects of BICD2 on ferroptosis, our data thus provide new insight into BICD2 action in the process of nephrolithiasis and other inflammatory diseases.

As the cargo adaptor, BICD2 predominantly localizes in cytoplasm and regulate the Golgi-endoplasmic reticulum transport under quiescent condition [22]. Here, we identify an inducible interaction between STAT1 and BICD2 upon exposure to ferroptosis agonist. We also find that BICD2 facilitates translocation of STAT1^Y701F^ from cytoplasm to nuclear following IFNγ stimulation. Notably, this BICD2-mediated nuclear accumulation of STAT1^Y701F^ was dependent on IFNγ stimulation, little nuclear STAT1^Y701F^ was detected in BICD2-expressing cells under quiescent condition. Given that IFNγ stimulation elicits little effects on STAT1^Y701F^, we thus hypothesize that IFNγ signaling may activate BICD2, which acts as an alternative pathway for promotion of STAT1 nuclear translocation.

In addition to ubiquitination, BICD2 has been reported to be phosphorylated on the serine residue S615 by vemurafenib [28]. Vemurafenib was first developed against lung cancers with the mutated form of the serine/threonine kinase BRAF, BRAF^V600E^ [33]. Along with chemotherapy, the newer targeted therapeutics can also cause kidney dysfunction through on and off-target mechanisms. Interestingly, recent study reveals that vemurafenib can trigger tubular damage and acute kidney injury [34]. Moreover, they find that kidney toxicity is BRAF-independent, while the underlying mechanism is partly unknown. Our present study shows that vemurafenib treatment triggers BICD2 phosphorylation and impairs its association with VHL, which in turn restricts BICD2 ubiquitination and blocks its proteasome degradation. Increased expression of BICD2 thereby enhances CaOx-induced oxidative stress and causes severe cell death and kidney injury. Our data thus uncover BICD2 as the potential target for the treatment of vemurafenib-induced nephrotoxicity.

In summary, our data uncover the critical role of VHL/BICD2/STAT1 axis in crystal-induced kidney injury and its related inflammatory damage. Through triggering BICD2 degradation, VHL blocks STAT1 nuclear accumulation and maintains the expression of SLC3A2 and SLC7A11 in renal epithelial cells, eventually reducing cell sensitivity to ferroptosis. Reciprocally, depletion of VHL or supplementation of the BRAF inhibitor Vemurafenib upregulates BICD2 expression, consequently promoting CaOx-induced lipid peroxidation and ferroptosis. Thus, our identification of the immunosuppressive role of VHL provides a potential therapeutic target for treatment and prevention of renal inflammation and drug-induced nephrotoxicity.

## Materials and methods

### Mice

*Vhl* ^+/mu^ mice (C57BL/6J background) were generated by CRISPR-Cas9-mediated gene editing. A gene-targeting construct containing the ATC(I) to TTC(F) missense mutation and neomycin-resistant gene flanked by *loxP* sites was electroporated into murine embryonic stem cells (ESCs). G418-resistant clones were identified by PCR and two positive clones were injected into blastocysts collected from C57BL/6J mice independently to achieve chimeras. The chimeras were subsequently crossed with EIIA-Cre transgenic female mice to remove the *loxP*-flanked neo-resistant gene (Neo). The resulting neo-deleted *Vhl* ^+/mu^ mice were genotyped by PCR with specific primers: Forward- TCTAGCCTTCTAACCCAGGTTGTCC, Reverse- GCCACAGCATCATTTTTACTTTCCA. All animals were maintained under a specific pathogen-free condition. All animal experiments were approved by the Ethics Committee of Peking University Health Science Center (LA2021487).

### Cell lines

Human embryonic kidney (HEK) 293T (HEK-293T) cells, 786-O, human kidney-2 (HK-2) cells were from American Type Culture Collection (ATCC) and authenticated by short tandem repeat (STR) profiling. HEK-293T cells were cultured in DMEM supplemented with 10% FBS plus 1% penicillin–streptomycin in a humidified atmosphere of 5% CO_2_. 786-O and HK-2 cells were cultured in RPMI-1640 supplemented with 10% FBS plus 1% penicillin–streptomycin in a humidified atmosphere of 5% CO2.

### Constructs

All constructs used for this study were prepared by standard molecular biology techniques and coding sequences entirely verified. All truncations and mutants were constructed by standard molecular biology techniques and confirmed by sequencing.

### Reagents

The reagents used in this study were as follow: Z-VAD-FMK, Erastin, RSL3, ferrostatin-1 (Fer-1), Deferoxamine (DFO), Vemurafenib, Selleck; H_2_O_2_, DMSO, Calcium Oxalate Monohydrate (COM), Sigma; Necrostatin-1 (Nec-1), abcam; Puromycin, ACROS; Cycloheximide (CHX), Biorbyt; MG132, Calbiochem; Recombinant human IFN-γ, Peprotech; Glyoxylic Acid (GA), TCI; Streptavidin magnetic beads, Beaverbio.

### CaOx nephrocalcinosis mouse model

For induction of CaOx nephrocalcinosis, 6–8-week-old female *Vhl* ^+/mu^ and wild-type (WT) mice were received intraperitoneal injection with 45 mg/kg of glyoxylate (glyoxylic acid, GA) (TCI, G0366) every day for 7 days and body weights were recorded every day.

### Histological and biochemical analyses

Mouse kidney tissues were fixed with formalin, embedded with paraffin and cut into 4 μm sections. For detection of kidney CaOx crystals, sections were stained using the von Kossa staining method (BestBio, BB-44711) following the manufacturer’s instructions. To assess kidney tissue damage, sections were stained with periodic acid-Schiff (PAS) (Beyotime, C0142S) following the manufacturer’s instructions. For haematoxylin-eosin (H&E) staining, sections were stained following a standard histopathological protocol. For detecting the expression of VHL, sections were transferred in antigen retrieval solution (Tris-EDTA, pH 6.0), after deparaffinization, blocking of endogenous peroxidase. Thereafter, sections were incubated overnight at 4 °C with anti-VHL antibody (Abclonal, A0377) and then detected using the Envision Detection System (Gene Tech, GK600705) according to the manufacturer’s instructions. All images were acquired using an Olympus IX51 microscope.

The levels of serum creatinine (SCR) were detected using Creatinine Assay Kit (Nanjing Jiancheng, C011-2-1), the levels of blood urea nitrogen (BUN) were detected by Urea Assay Kit (Nanjing Jiancheng, C013-2-1), the levels of blood uric acid (UA) were detected using Uric acid Assay Kit (Nanjing Jiancheng, C012-2-1). The levels of malondialdehyde (MDA) were detected using Lipid Peroxidation MDA Assay Kit (Beyotime, S0131S), the levels of reduced glutathione (GSH) were detected using GSH assay kit (Nanjing Jiancheng, A006-2-1), the levels of tissue calcium were detected using Calcium Colorimetric Assay Kit (Elabscience, E-BC-K103-M) and the levels of tissue iron were detected using Iron Colorimetric Assay Kit (Elabscience, E-BC-K139-M) according to the manufacturer’s instructions.

### Renal tubular injury score

To further evaluate the renal tubular injury, we analyzed the HE and PAS staining results according to previous studies [35, 36]. Briefly, the kidney damage was scored according to the following aspects: tubular dilation, tubular necrosis, denuded or ruptured tubular basement membranes, epithelial cell apoptosis, intraluminal cast formation, and brush border loss. We randomly selected 5 nonoverlapping high-power microscopic fields form every section and calculated the ratio of tubular injury. Grades 0–5 represented 0%, ≤10%, 11–25%, 26–45%, 46–75%, and ≥76% injury, respectively.

### Immunohistochemical score

The immunohistochemical score of VHL was performed using a semiquantitative system as demonstrated previously [25, 26]. Briefly, histological analyses were performed by combining the density and intensity of positive staining cells. Each slide was assigned a score: the score of cell staining multiplied by the score of staining intensity. The density classification of positive cells was itemized here below: 0, the number of positive cells <5%; 1, the number of positive cells 5–25%; 2, the number of positive cells 26–50%; 3, the number of positive cells 51–75%; 4, the number of positive cells >75%. The intensity of positive staining cells was itemized here below: 0, absence of staining; 1, light yellow staining; 2, brownish yellow staining; 3, brownish brown staining. The immunoreactivity of proteins was scored on the basis of the intensity and density of positive stained cells, and the scores were calculated and assessed by two independent investigators.

### Lentiviral packing and infection

To obtain VHL and BICD2 stably transfected cells, the sequences of VHL and BICD2 were cloned into pCDH-CMV-MCS-EF1-copGFP lentiviral vector respectively. HEK293T cells were transfected with psPAX2, pMD2.G and lentiviral constructs. Supernatants were collected at 48 h post-transfection. After passing through 0.45-µm filters, viruses were used to infect target cells supplemented with 8 μg/ml polybrene. Subsequently, GFP^+^ cells were sorted by flow cytometry.

For knockdown HIF1A, HIF2A and BICD2 in 786-O cells, shRNAs targeting *HIF1A* (1^#^: CGGCGAAGTAAAGAATCTGAA; 2^#^: GTGATGAAAGAATTACCGAAT), *HIF2A* (1^#^: GCCACAGCATGGACATGAAGT; 2^#^: GCATCATGTGTGTCAACTACG), *BICD2* (1^#^: CCAGGTGTGACGAGTACATTA; 2^#^: GCCAACCTGAAGAGCAAGTAT) were cloned into pLKO.1 plasmid. 786-O cells were transfected with psPAX2, pMD2.G and lentiviral constructs. After viral infection, positive cells were selected by 2 μg/ml puromycin.

### Quantitative real-time PCR

Total RNA was extracted with Trizol reagent (Invitrogen) and reverse transcribed into cDNA with GoScriptTM Reverse Transcription System (Promega) according to the manufacturer’s protocol. qRT-PCR was performed using SYBR qPCR Master Mix (Vazyme) and ABI 7500 Detection System. All primers are listed in Table S1.

### Co-immunoprecipitation and immunoblot analysis

Cells were transfected with appropriate plasmids and lysed by co-immunoprecipitation lysis buffer (10% glycerol, 0.5% NP-40, 150 mM NaCl, 0.1 mM EDTA) supplemented with protease inhibitor cocktail (Roche). Cell lysates were incubated with the S-protein Agarose beads (Millipore, 69704) or indicated primary antibody and protein A/G agarose beads (Santa Cruz Biotechnology, sc-2003). The immunocomplexes were then washed by PBSN (PBS containing 0.1% NP-40) three times and subjected to SDS-Page. For subcellular fractionation, nuclear and cytoplasmic extracts were isolated with a nuclear-cytoplasmic extraction kit (Applygen, P1200) following the manufacturer’s protocol.

Antibodies used in this study were as follows: anti-VHL (Abcam, ab77262) for Western Blot, anti-VHL (Abclonal, A0377) for Immunohistochemistry, anti- EPAS-1/HIF-2 alpha (Santa Cruz Biotechnology, sc-13596), anti-BICD2 (Abcam, ab237616), anti-GAPDH (TransGen Biotech, HC301-01), anti-β-Tubulin (ABclonal, AC021), anti-β-Actin (ABclonal, AC004), anti-HDAC1 (ABclonal, A19571), anti-FLAG (Sigma, F3165), anti-HA (Santa Cruz Biotechnology, sc-7392) and anti-GFP (MBL, 598).

### Ubiquitination assay

HEK293T cells were transfected with appropriate plasmids. 24 hours later, cells were treated with 10 μM of the proteasome inhibitor MG132 (Calbiochem) for 10 h. Cells were harvested and extracted in 100 µL of co-immunoprecipitation lysis buffer mentioned above supplemented with 1% SDS. Cell extracts were heat-denatured for 5 min at 100°C and diluted with co-immunoprecipitation lysis buffer containing protease inhibitors (Roche) and 20 μM MG132 to an SDS concentration of ≤0.1%. Diluted cell lysates were sonicated and centrifuged to clarify, followed by immunoprecipitation as described above.

### Protein half-life assay

For the half-life assay, HEK293T cells were transfected with appropriate plasmids. 24 hours later, cells were treated with the protein synthesis inhibitor cycloheximide (Biorbyt, 200 μg/ml) with or without the proteasome inhibitor MG132 (Calbiochem) for the indicated durations before collection. Cells were harvested and lysed for immunoblot analysis.

### TurboID-based proximity labeling technology

TurboID-based proximity labeling assay was performed as previously described [37]. In brief, HEK293T cells were transfected with the VHL-TurboID or Mock-TurboID plasmid. After 24 h, biotin was added at a final concentration of 500 μM for 10 min. Cells were harvested and lysed with RIPA lysis buffer (50 mM Tris, 150 mM NaCl, 0.1% SDS, 0.5% sodium deoxycholate, 1% Triton X-100) with protease inhibitor cocktail (Roche) at 4 °C for 30 min. The lysates were centrifuged to clarify and the supernatants were incubated with Streptavidin magnetic beads (Beaverbio, 22305-1) overnight at 4 °C. Subsequently, the beads were washed twice with RIPA lysis buffer, once with 1 M KCl, once with 0.1 M Na_2_CO_3_, once with 2 M urea in 10 mM Tris–HCl (pH 8.0), and twice with RIPA lysis buffer. Finally, biotinylated proteins were eluted by boiling the beads in 100 μl of elution buffer (55 mM pH 8.0 Tris-HCl, 0.1% SDS, 6.66 mM DTT, 0.66 mM biotin) for 10 min at 100 °C. The eluted samples were subjected to NuPAGE 4–12% gel (Invitrogen) and sliver staining (Pierce, 24612). The excised gel segments were subjected to mass spectrum analysis.

### Flag pull-down assay

FLAG pulldown assay was performed as previous study [38]. Briefly, HEK293T cells were transfected with plasmid expressing mock or FLAG-tagged BICD2. 24 hours later, cells were harvested and lysed with co-immunoprecipitation lysis buffer at 4 °C for 30 min. The lysates were centrifuged to clarify and the supernatants were enriched with anti-FLAG M2 beads (Sigma, F2426) at 4 °C overnight. The binding components were eluted with 3×FLAG peptide (Sigma, F4799). The samples were subjected to NuPAGE 4–12% gel (Invitrogen) and sliver staining (Pierce, 24612). The excised gel segments were subjected to mass spectrum analysis.

### Mass spectrum analysis

Mass spectrum analysis was performed as previously described [39]. Briefly, after sliver staining of a gel, the gel was excised and subjected to in-gel trypsin digestion and dried. Peptides were dissolved in 10 μL 0.1% formic acid and auto-sampled directly onto a 100 μm × 10 cm fused silica emitter made in our laboratory packed with reversed-phase ReproSil-Pur C18-AQ resin (3 μm and 120 Å; Ammerbuch, Germany). Samples were then eluted for 50 min with linear gradients of 5–32% acetonitrile in 0.1% formic acid at a flow rate of 300 nl/min. Mass spectrometry data were acquired with an LTQ Orbitrap Elite mass spectrometer (Thermo Fisher Scientific) equipped with a nanoelectrospray ion source (Proxeon Biosystems). Fragmentation in the LTQ was performed by collision-induced dissociation (normalized collision energy, 35%; activation Q, 0.250; activation time, 10 ms) with a target value of 3000 ions. The raw files were searched with the SEQUEST engine against a database from the UniProt protein sequence database.

### Preparation of lymphocytes

To isolate lymphocytes infiltrating in the kidney, minced tissues were incubated with digestion solution containing 0.5 mg/ml collagenase D (Roche, 11088866001) and 25 μg/ml DNase I (Sigma, DN25) at 37 °C for 45 min with slow rotation. Then the tissues were grinded, and filtered through a 75 μm strainer. Mononuclear cells were isolated through 40/80% Percoll (GE Healthcare) by gradient centrifuging 800 × *g* for 20 min. Cells at the inter-layer were collected and counted for further operation.

### Flow cytometry

To analyze cell surface maker expression, cells were incubated with specific antibodies for 30 min at room temperature. The flow cytometry analyzer (BD Biosciences) were used for acquiring the cells. The FACS data were analyzed with FlowJo v10 software.

Following antibodies were used: anti-CD4 (GK1.5, 100408, 1:250), anti-CD8a (53-6. 7, 100712, 1:250), anti-CD45 (30-F11, 03113, 1:250), Anti-CD3ε (145-2C11, 100326, 1:250), anti-CD11b (M1/70, 101206, 1:250), anti-F4/80 (BM8, 123116, 1:250), anti-Ly6G (1A8, 127654, 1:250), anti-Ly6C (HK1.4, 128008, 1:250) (all from Biolegend); anti-CD19(eBio1D3, 25-0193-82, 1:250), anti-NK1.1 (PK136, 11-5941-82, 1:250) (all from eBioscience).

### LDH, ATP and CCK-8 assay

Relevant cells were seeded in 96-well plates and treated as indicated. LDH Cytotoxicity Assay Kit (Beyotime Biotech, C0017) was used to determine lactate dehydrogenase (LDH) release and cytotoxicity, ATP Assay Kit (Beyotime Biotech, S0026) was used to determine adenosine triphosphate (ATP) release and a Cell counting Kit-8 (CCK-8, Dojindo) assay was used to detect the cell viability. All assays were performed according to the manufacturer’s instructions.

### Annexin V/7-AAD staining

Relevant cells were seeded in 6-well plates and treated as indicated. Annexin V/7-AAD staining was performed using Annexin V-PE/7-AAD Apoptosis Detection Kit (Vazyme, A213), following the manufacturer’s instructions.

### Detection of ROS production

Reactive Oxygen Species Assay Kit (Beyotime Biotech, S0033) was used to detect generation of ROS in cultured cells. Briefly, cells were washed with PBS twice, followed by staining with 10 μM DCFDA for 20 min at 37 °C. After washing with PBS, stained cells were subjected to flow cytometry analysis.

### Quantification and statistical analysis

Prism GraphPad software v8.0.2 was used for statistical analysis. The statistical significance between different groups were calculated using a two-tailed Student’s t-test. *P* < 0.05 was considered significant. All experiments were independently replicated at least three times and similar results were generated.

### Supplementary information

Supplementary Information

Original data files

Checklist

## Data Availability

The RNA-seq data generated in this study have been deposited in the GEO database with the accession code GSE208528 and GSE209996. The mass spectrometry proteomics data have been deposited to the ProteomeXchange Consortium via the PRIDE [40] partner repository with the dataset identifier PXD042830. S615 mutation of BICD2 is from PhosphoSitePlus database (PSP, https://www.phosphosite.org). The data used to support the findings of this study are available from the corresponding author upon request.
