# Supplementary material for: Critical role of VHL/BICD2/STAT1 axis in crystal-associated kidney disease
Source: Cell Death Dis. 2023 Oct 13;14(10):680. doi: 10.1038/s41419-023-06185-1 (PMC10575931; doi:10.1038/s41419-023-06185-1)
Supplement: Supplementary file 1 — Supplementary Information [file 41419_2023_6185_MOESM1_ESM.docx]

Supplementary information

**Critical role of VHL/BICD2/STAT1 axis in crystal-associated kidney disease**

Wenyan Hao^1^, Hongxian Zhang^2^, Peng Hong^2^, Xin Zhang^1^, Xuyang Zhao^1^, Lulin Ma^2^, Xiaoyan Qiu^3^, Hao Ping^4,5*^, Dan Lu^1*^ and Yuxin Yin^1*^

^1^Institute of Systems Biomedicine, Department of Immunology, School of Basic Medical Sciences, NHC Key Laboratory of Medical Immunology, Beijing Key Laboratory of Tumor Systems Biology, Peking University, Beijing 100191, P.R. China.

^2^Department of Urology, Peking University Third Hospital, Beijing 100191, P.R. China.

^3^Department of Immunology, School of Basic Medical Sciences, Peking University, Beijing 100191, P.R. China.

^4^Department of Urology, Beijing Tongren Hospital, Capital Medical University, Beijing 100730, P.R. China.

^5^Beijing Advanced Innovation Center for Big Data-Based Precision Medicine, Beihang University and Capital Medical University, Beijing Tongren Hospital, Beijing 100730, P.R. China.

This PDF file includes:

Figure S1 to S9

Table S1

**
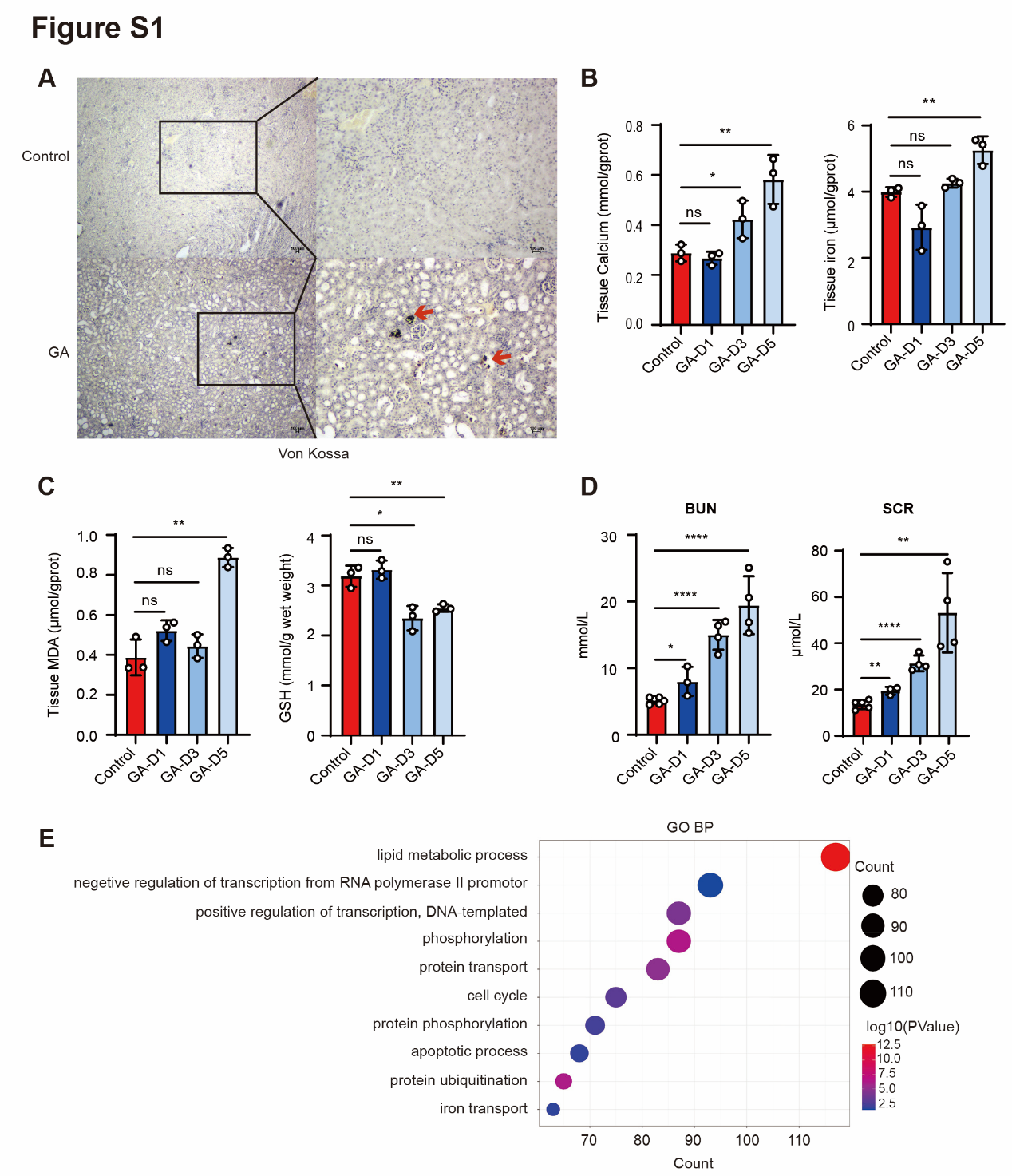
**

**Figure S1: Construction of murine model of CaOx nephrocalcinosis**

**(A-E)** 6-8 weeks old wild-type mice were received intraperitoneal injection with either saline (control) or 60 mg/kg of glyoxylate (glyoxylic acid, GA) every day. Mice were harvested respectively on day 1, 3, 5, 7 after the first injection.

**(A)** The kidneys were subjected to von Kossa staining on day 7 after the first injection. The image is representative of five mice with similar results. The red arrow indicates kidney CaOx crystals. Scale bar: 100 μm.

**(B)** Assessment of the levels of calcium and iron in kidney tissues after GA injection (n = 3 mice, mean ± SD, ns, not significant, **P* (Tissue Calcium) = 0.0467, ***P* (Tissue Calcium) = 0.0079; ***P* (Tissue Iron) = 0.0079).

**(C)** Assessment of the levels of malondialdehyde (MDA) and reduced glutathione (GSH) in kidney tissues after GA injection (n = 3 mice, mean ± SD, ns, not significant; ***P* (Tissue MDA) = 0.0010; **P* (GSH) = 0.0108, ***P* (GSH) = 0.0081).

**(D)** Assessment of the levels of blood urea nitrogen (BUN) and serum creatinine (SCR) of mice after GA injection (n = 3-6 mice, mean ± SD, ns, not significant; **P* (BUN) = 0.0140; ***P* (SCR-GA-D1) = 0.0045, ***P* (SCR-GA-D5) = 0.0012, *****P* < 0.0001).

**(E)** Differentially expressed genes that were up-regulated in kidney from GA-treated mice versus control mice were used for enrichment analysis with the gene ontology (GO) database.

Statistical significance was assessed by two-tailed unpaired Student’s t test, **P* < 0.05; ***P* < 0.01; ****P* < 0.001; *****P* < 0.0001. Each data point refers to the number of mice per cohort used per experiment, in *vivo* experiments were repeated three times reproducibly, data shown is from one repeat.


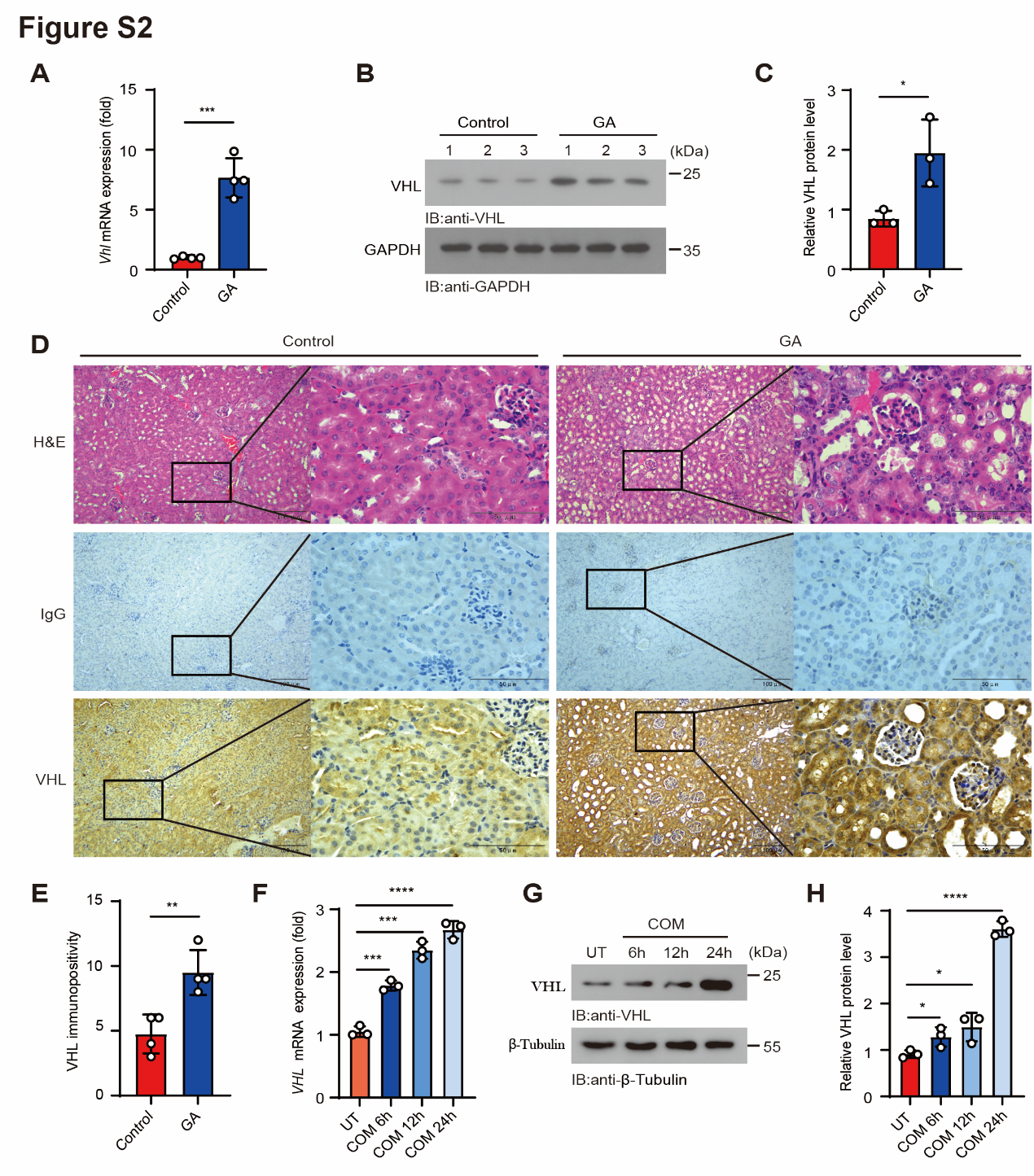


**Figure S2: VHL is upregulated during nephrolithiasis**

**A-D** 6-8 weeks old wild-type mice were received intraperitoneal injection with either saline (control) or 60 mg/kg of glyoxylate (glyoxylic acid, GA) every day. The kidneys were collected on day 5 after the first injection.

**A** The mRNA level of *Vhl* in kidney was determined by qRT-PCR assay (n = 4 mice, mean ± SD, ****P* = 0.0002).

**B** Immunoblot analysis of VHL protein expression in kidney. An anti-VHL antibody was used (n = 3 mice).

**C** Gray values of VHL relative to GAPDH were determined by ImageJ software, using for the histogram. (n = 3 mice, mean ± SD, **P* = 0.0294).

**D** Immunohistochemical analysis of VHL protein expression in kidney. H&E staining and isotype IgG antibody served as control.

**E** Semiquantitative evaluation of VHL immunopositivity (n = 4 mice; mean ± SD, **P = 0.0060).

**F-H** HK-2 cells were treated with 100 μM COM for indicated hours. UT, untreatment.

**F** qRT-PCR analysis of *VHL* mRNA level was determined (n = 3 cell cultures, mean ± SD, ****P* (COM 6h) = 0.0004, ****P* (COM 12h) = 0.0001, *****P* < 0.0001).

**G** Immunoblot analysis of VHL protein expression level were examined.

**H** Gray values of VHL relative to β-Tubulin were determined by ImageJ software, using for the histogram (n=3 three independent experiments, mean ± SD, **P* (COM 6h) = 0.0474, **P* (COM 12h) = 0.0335, *****P* < 0.0001).

Statistical significance was assessed by two-tailed unpaired Student’s t test, **P* < 0.05; ***P* < 0.01; ****P* < 0.001, *****P* < 0.0001 (**A, C, E, F and H**). Experiments were repeated three times reproducibly, data shown is from one repeat. For *in vivo* experiments, each data point refers to the number of mice per cohort used per experiment; for *in vitro* experiments, each data point refers to an individual cell culture within the experiment.


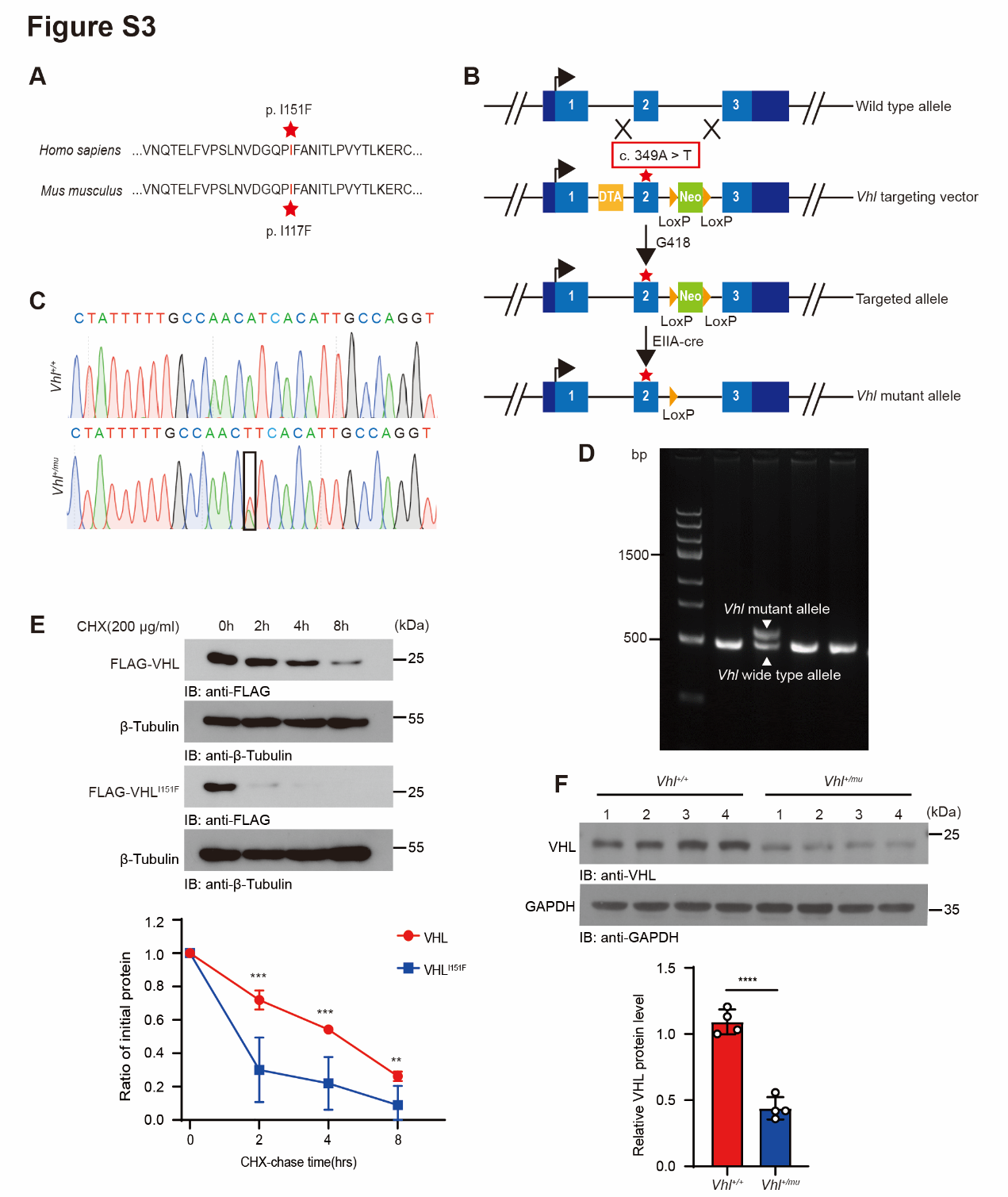


**Figure S3: The generation of *Vhl^+/mu^* mice**

**(A)** The alignments of VHL protein sequences of *Homo sapiens* and *Mus musculus.* Missense mutation I117F in mouse is conserved with the I151F mutation in human.

**(B)** Construction strategy for *Vhl^+/mu^* mice.

**(C)** *Vhl* allelic sequences of *Vhl^+/+^* and *Vhl^+/mu^* mice*.*

**(D)** Genotyping of *Vhl^+/+^* and *Vhl^+/mu^* mice. Mutant allele is indicated at the top and wild-type allele is indicated at the bottom.

**(E)** Half-life analysis of VHL and VHL^I151F^ in HEK293T cells. Cells transfected with FLAG-tagged VHL or VHL^I151F^ plasmid were treated with 200 μg/ml cycloheximide (CHX) for indicated times for immunoblot analysis (up), and gray values of VHL and VHL^I151F^ relative to β-Tubulin were determined by ImageJ software, using for the line chart (down) (n = 3 independent experiments, mean ± SD, ****P* (2h) = 0.000866, ****P* (4h) = 0.000960, ***P* (8h) = 0.003295).

**(F)** Immunoblot analysis of VHL protein expression in *Vhl^+/+^* and *Vhl^+/mu^* mice using anti-VHL antibody (up). Gray values of VHL relative to GAPDH were determined by ImageJ software, using for the histogram (down) (n = 4 mice, mean ± SD, *****P* < 0.0001).

Statistical significance was assessed by two-tailed unpaired Student’s t-test, ***P* < 0.01; ****P* < 0.001; *****P* < 0.0001 (**E and F**). The immunoblot images shown are from one repeat. The line chart is from 3 independent studies (**E**). Each data point refers to the number of mice per cohort used per experiment, in *vivo* experiments were repeated three times reproducibly, data shown is from one repeat (**F**).


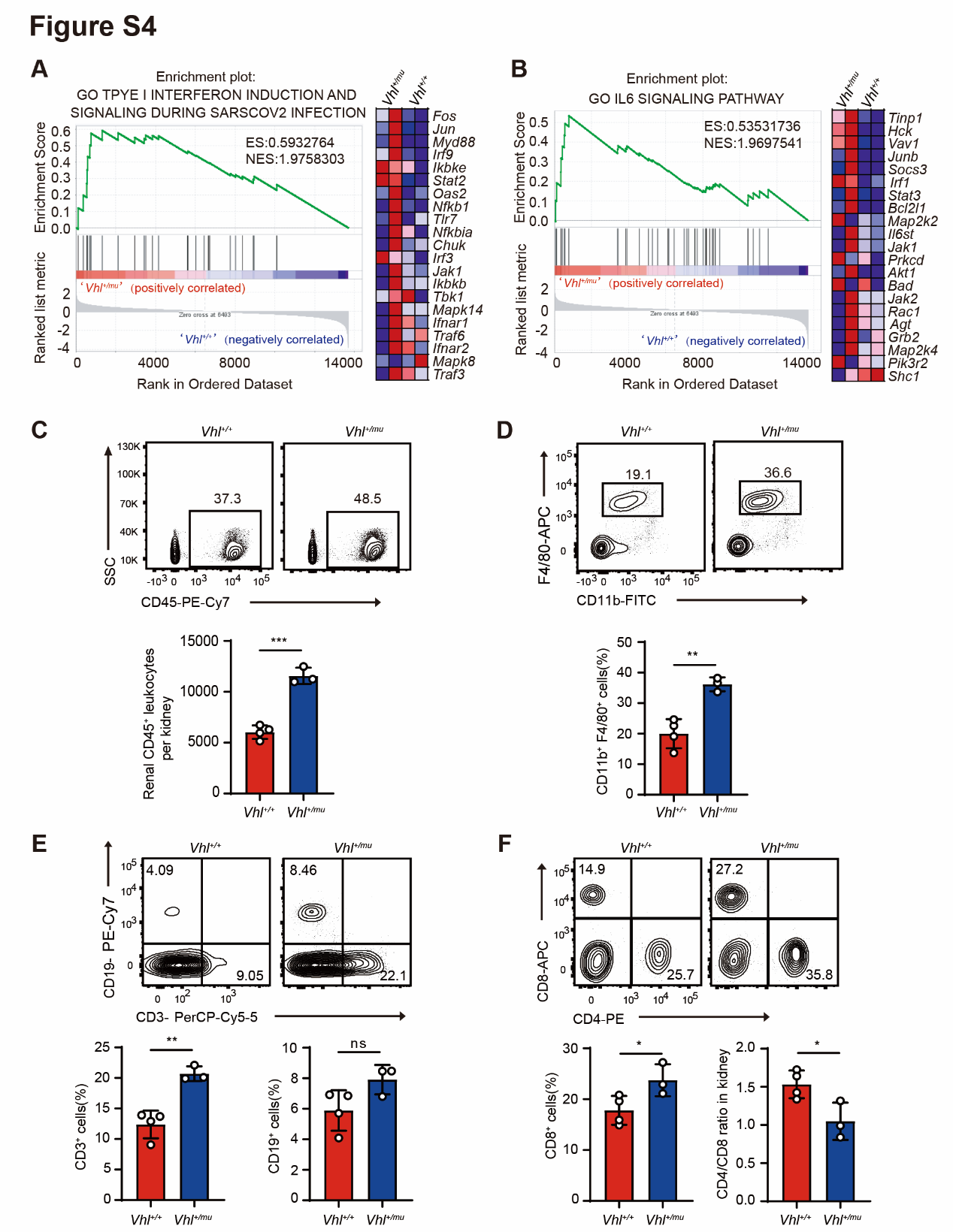


**Figure S4: VHL deficiency exacerbates inflammatory damage during nephrolithiasis.**

6-8 weeks old *Vhl^+/+^* and *Vhl^+/mu^* mice were received intraperitoneal injection with 45 mg/kg of glyoxylate (glyoxylic acid, GA) every day for 7 days to induce CaOx nephrocalcinosis. The kidneys were collected and subjected to RNA-sequencing as well as flow cytometry.

**(A-B)** GSEA of the differentially expressed genes in *Vhl^+/+^* and *Vhl^+/mu^* mice after GA injection. ES, enrichment score; NES, normalized enrichment score.

**(C)** Flow cytometry analysis of the percentages and numbers of CD45^+^ leukocytes cells from the kidney of *Vhl^+/+^* and *Vhl^+/mu^* mice after GA injection (*Vhl^+/+^*, n = 4 mice; *Vhl^+/mu^*, n = 3 mice; mean ± SD., ****P* = 0.0002).

**(D-F)** Flow cytometry analysis of the percentages of CD11b^+^ F4/80^+^ cells (D), CD3^+^ cells, CD19^+^ cells (E), CD4^+^ cells, CD8^+^ cells (F) from the kidney of *Vhl^+/+^* and *Vhl^+/mu^* mice after GA injection. (*Vhl^+/+^*, n = 4 mice; *Vhl^+/mu^*, n = 3 mice; mean ± SD, ns, not significant, ***P* (CD11b^+^ F4/80^+^ cells) = 0.003112; ***P* (CD3^+^ cells) = 0.002360; **P* (CD8^+^ cells) = 0.047009, **P* (CD4/CD8 ratio) = 0.0289).

Statistical significance was assessed by two-tailed unpaired Student’s t-test, **P* < 0.05; ***P* < 0.01; ****P* < 0.001. Each data point refers to the number of mice per cohort used per experiment, in *vivo* experiments were repeated three times reproducibly, data shown is from one repeat (**C -F**).

**
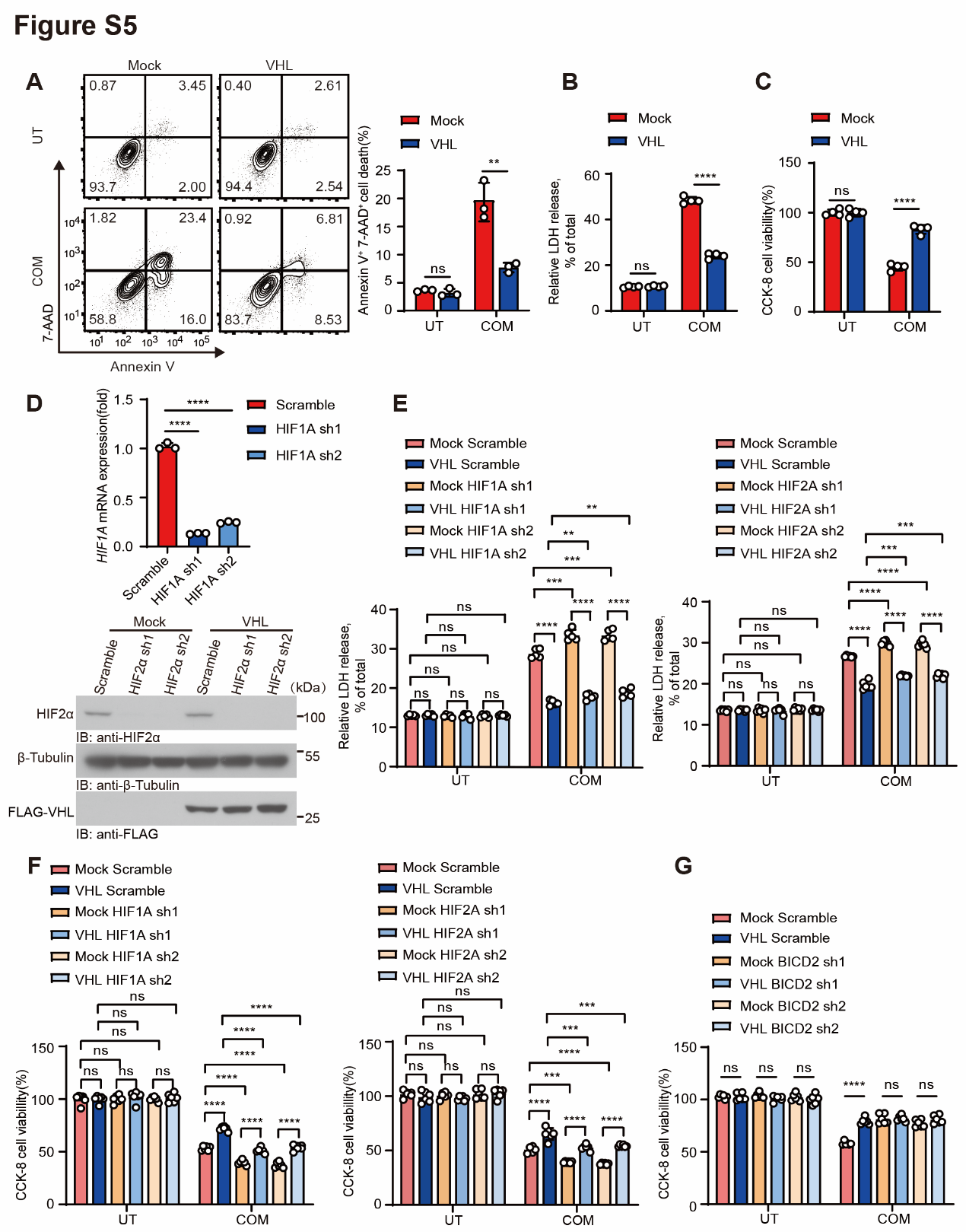
**

**Figure S5: VHL protects kidney in a HIF-independent manner.**

**(A-C)** Mock or VHL stably-expressing 786-O cells were treated with 200 μM COM for 24 hours. UT, untreatment.

**(A)** The death cells were assessed by flow cytometric analysis of Annexin V/7-AAD staining. The percentages of Annexin V^+^ 7-AAD^+^ cells were analyzed (right) (n = 3 cell cultures, mean ± SD, ns, not significant, ***P* = 0.004622).

**(B)** LDH release was measured (n = 4 cell cultures, mean ± SD, ns, not significant, *****P* < 0.0001).

**(C)** The cell viability was detected by Cell counting Kit-8 (CCK-8). (n = 4 cell cultures, mean ± SD, ns, not significant, *****P* < 0.0001).

**(D)** The effectiveness of *HIF1A* and *HIF2A* knockdown in 786-O cells were assessed by qRT-PCR assay and immunoblot assay, respectively. (n = 3 cell cultures, mean ± SD, *****P* < 0.0001).

**(E-G)** Endogenous *HIF1A*, *HIF2A* or *BICD2* were silenced respectively in Mock and VHL stably-expressing 786-O cells by shRNA assay. Cells were treated with 200 μM COM for 24 hours. UT, untreatment.

**(E)** LDH release was detected. (n = 5 cell cultures, mean ± SD, ns, not significant, ****P* = 0.000418, ****P* = 0.000391, ***P* = 0.008253, ***P* = 0.002525 (left panel, from down to up); ****P* = 0.000219, ****P* = 0.000484 (right panel, from down to up); *****P*< 0.0001).

**(F** **and** **G)** The cell viability was detected by Cell counting Kit-8 (CCK-8). (n = 6 cell cultures, mean ± SD, ns, not significant, ****P* = 0.000218, ****P* = 0.000547, ****P* = 0.000697 (right panel, from down to up); *****P* < 0.0001).

Statistical significance was assessed by two-tailed unpaired Student’s t-test, **P* < 0.05; ***P* < 0.01; ****P* < 0.001; *****P* < 0.0001. Experiments were repeated three times reproducibly, data shown is from one repeat.


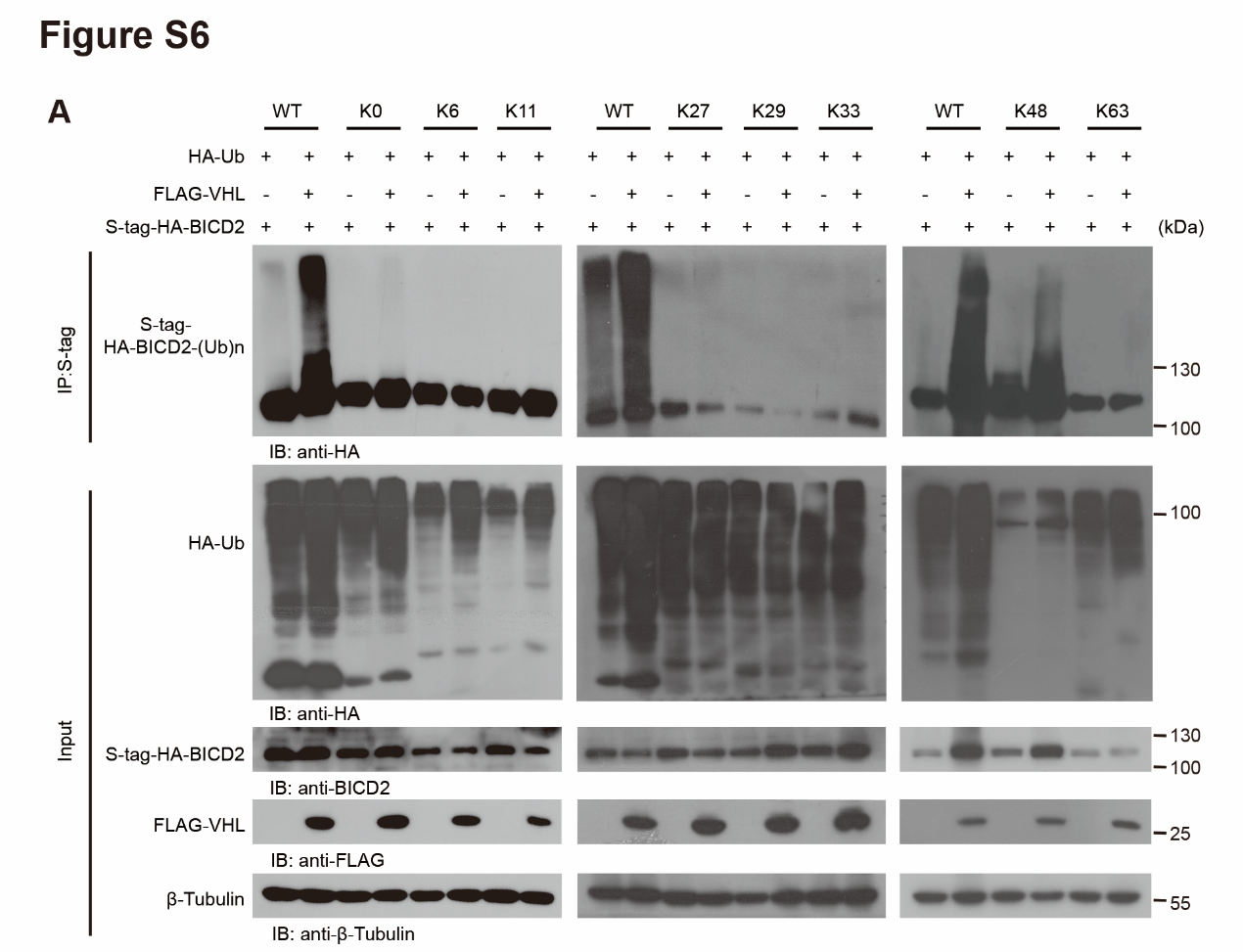


**Figure S6: VHL triggers K48-linked poly-ubiquitination of BICD2.**

**(A)** The ubiquitination linkage assay of BICD2. HEK293T cells were transfected with BICD2, VHL and the indicated vectors encoding WT or mutant ubiquitin, followed by treatment with 10 μM MG132 for 10 hours before collection. The whole-cell lysate was subjected to pulldown with S-protein Agarose beads and immunoblot. WT ubiquitin was used as a positive control. Experiments were repeated three times reproducibly, data shown is from one repeat.


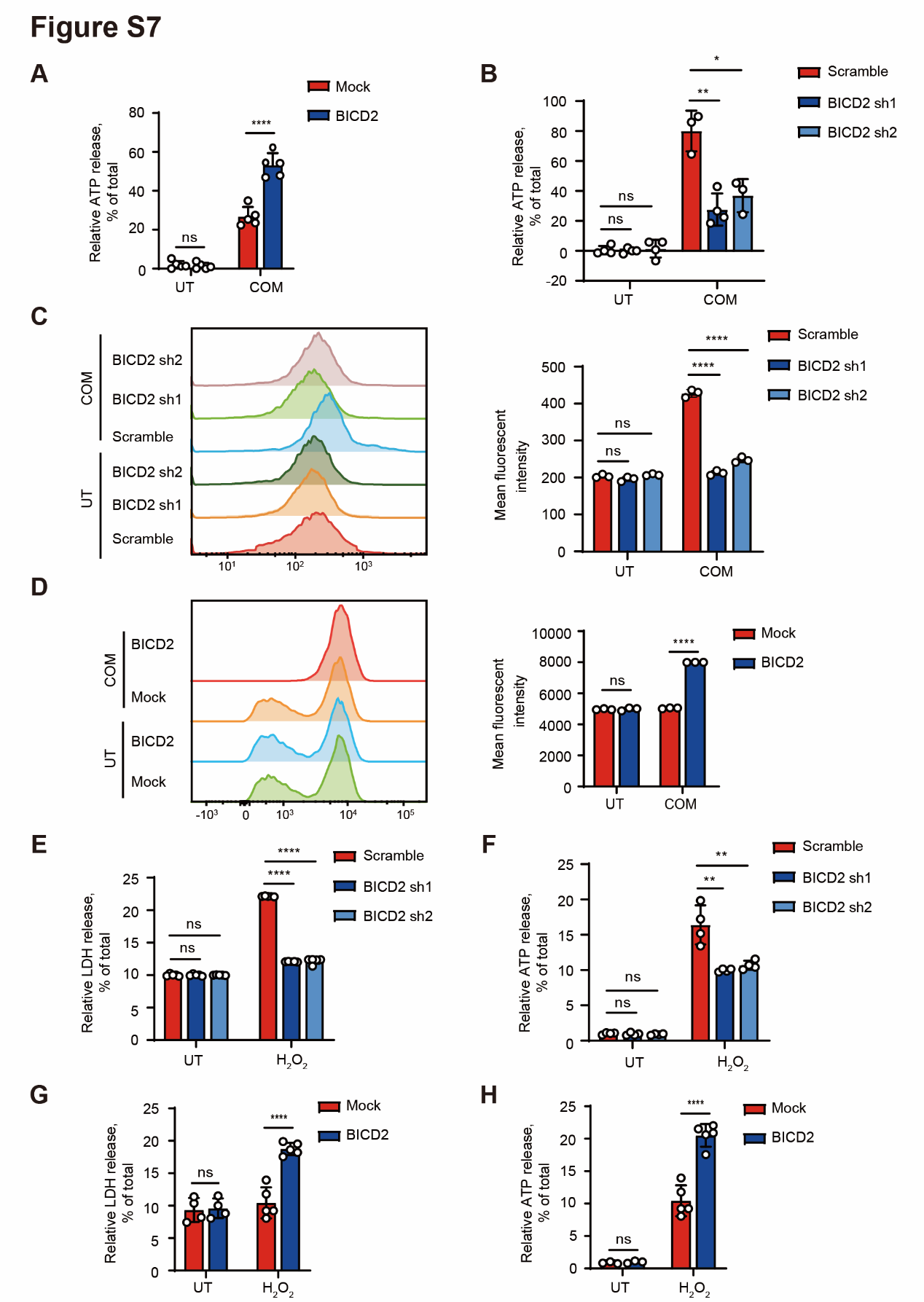


**Figure S7: BICD2 increases cell sensitivity to oxidative stress.**

**(A-B)** Mock or BICD2 stably-expressing HK-2 cells **(A)**, BICD2-deficient or control 786-O cells **(B)** were treated with 200 μM COM for 24 hours. UT, untreatment. The supernatants were collected and used to ATP release assay.

**(A)** Assessment of the ATP release from Mock or BICD2 stably-expressing HK-2 cells (n = 5 cell cultures, mean ± SD, ns, not significant, *****P* < 0.0001).

**(B)** Measurement of the ATP release from BICD2-deficient or control 786-O cells (n = 3-5 cell cultures, mean ± SD, ns, not significant, ***P* = 0.002239, **P* = 0.012963).

**(C-D)** BICD2-deficient or control 786-O cells **(C)**, Mock or BICD2 stably-expressing HK-2 cells **(D)** were treated with 200 μM COM for 2 hours. UT, untreatment. Intracellular ROS production was measured by DCFDA staining using for flow cytometry analysis. Mean fluorescence intensity of DCFDA was quantified using Flowjo software (right).

**(C)** Assessment of intracellular ROS production from BICD2-deficient or control 786-O cells (n = 3 cell cultures, mean ± SD, ns, not significant, *****P* < 0.0001).

**(D)** Measurement of intracellular ROS production from Mock or BICD2 stably-expressing HK-2 cells (n = 3 cell cultures, mean ± SD, ns, not significant, *****P* < 0.0001).

**(E-F)** BICD2-deficient or control 786-O cells were treated with 100 μM H_2_O_2_ for 2 hours. UT, untreatment. The supernatants were collected and used to LDH **(E)** and ATP **(F)** release assay.

**(E)** Assessment of LDH release from indicated cells (n = 5 cell cultures, mean ± SD, ns, not significant, *****P* < 0.0001).

**(F)** Assessment of extracellular ATP release from indicated cells (n = 4 cell cultures, mean ± SD, ns, not significant, ***P* = 0.003527, ***P* = 0.006870).

**(G-H)** Mock or BICD2 stably-expressing HK-2 cells were treated with 100 μM H_2_O_2_ for 2 hours. UT, untreatment. The supernatants were collected and used to LDH **(G)** and ATP **(H)** release assay.

**(G)** Assessment of LDH release from indicated cells (n = 5 cell cultures, mean ± SD, ns, not significant, *****P* < 0.0001).

**(H)** Assessment of ATP release from indicated cells. (n = 5 cell cultures, mean ± SD, ns, not significant, *****P* < 0.0001).

Statistical significance was assessed by two-tailed unpaired Student’s t-test, **P* < 0.05; ***P* < 0.01; ****P* < 0.001; *****P* < 0.0001. Each data point refers to an individual cell culture within the experiment, experiments were repeated at least three times reproducibly, data shown is from one repeat.


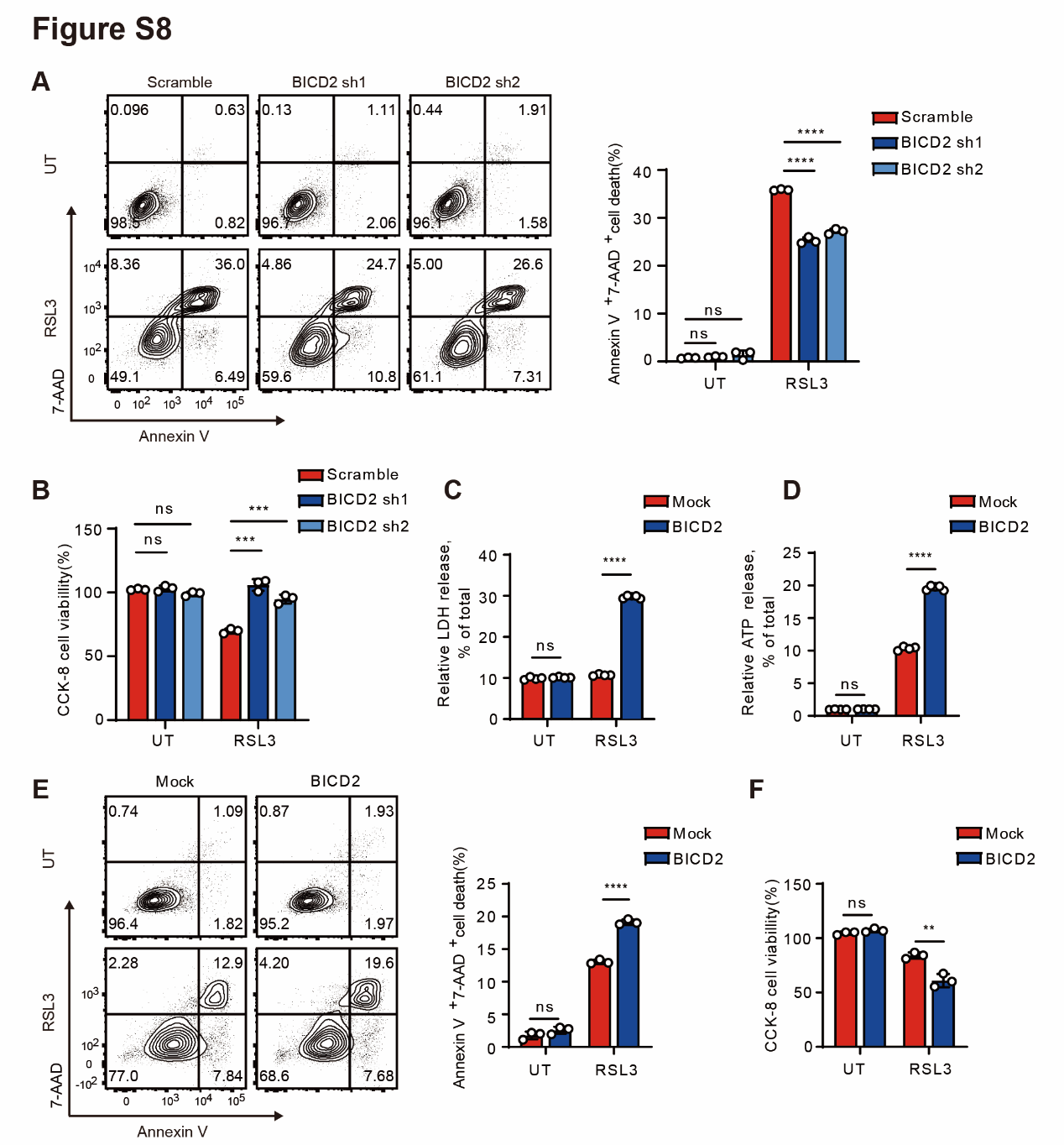


**Figure S8: BICD2 increases cell sensitivity to ferroptosis.**

**(A-B)** BICD2-deficient or control 786-O cells were treated with 0.4 μM ferroptosis agonist RSL3 for 6 hours. UT, untreatment.

**(A)** Flow cytometric analysis of dying cells using Annexin V/7-AAD staining. The percentages of dying cells (Annexin V^+^ 7-AAD^+^) were analyzed (right) (n = 3 cell cultures, mean ± SD, ns, not significant, *****P* < 0.0001).

**(B)** The CCK-8-based cell viability of BICD2-deficient or control 786-O cells. (n = 3 cell cultures, mean ± SD, ns, not significant, ****P* = 0.000226, ****P* = 0.000384).

**(C-F)** Mock or BICD2 stably-expressing HK-2 cells were treated with 0.4 μM ferroptosis agonist RSL3 for 6 hours. UT, untreatment.

**(C)** Assessment of LDH release from Mock or BICD2 stably-expressing HK-2 cells (n = 4 cell cultures, mean ± SD, ns, not significant, *****P* < 0.0001).

**(D)** ATP release assay of Mock or BICD2 stably-expressing HK-2 cells (n = 4 cell cultures, mean ± SD, ns, not significant, *****P* < 0.0001).

**(E)** Flow cytometric analysis of dying cells using Annexin V/7-AAD staining. The percentage of Annexin V^+^ 7-AAD^+^ cells were analyzed (right) (n = 3 cell cultures, mean ± SD, ns, not significant, *****P* < 0.0001).

**(F)** The CCK-8-based cell viability of Mock or BICD2 stably-expressing HK-2 cells (n = 3 cell cultures, mean ± SD, ns, not significant, ***P* = 0.004111).

Statistical significance was assessed by two-tailed unpaired Student’s t-test, ***P* < 0.01; ****P* < 0.001; *****P* < 0.0001. Each data point refers to an individual cell culture within the experiment, experiments were repeated at least three times reproducibly, data shown is from one repeat.


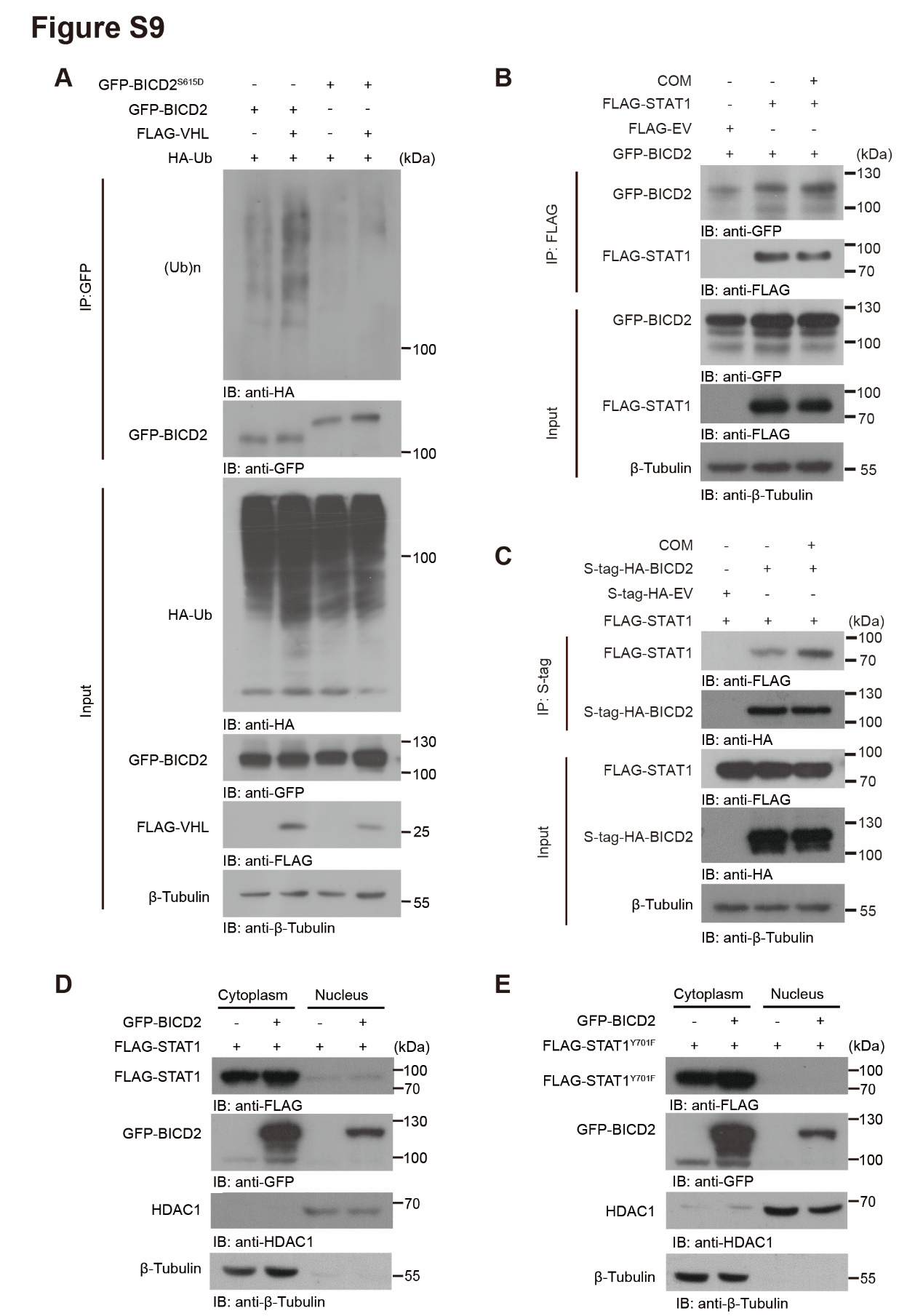


**Figure S9：The effects of various stimulations on the VHL/BICD2/STAT1 axis.**

**(A)** HEK293T cells were transfected with the indicated plasmids (above lanes), followed by 10 μM MG132 treatment for 10 hours before collection. Cell lysates were immunoprecipitated with anti-GFP antibody. The immunoprecipitated proteins were subjected to immunoblot analysis.

**(B-C)** HEK293T cells were transfected to express BICD2 and Mock or STAT1, with or without 100 μM COM treatment for 6 h before collection. Cell lysates were immunoprecipitated with anti-FLAG antibody **(B)** or S-protein Agarose beads **(C)**, followed by immunoblot analysis with anti-GFP antibody **(B)** or anti-FLAG antibody **(C)**.

**(D)** HEK293T cells were co-transfected with FLAG-tagged STAT1 and Mock or GFP-tagged-BICD2 plasmid. Nuclear and cytoplasmic extracts were isolated and analyzed by immunoblot.

**(E)** HEK293T cells were co-transfected with vectors encoding FLAG-tagged STAT1^Y701F^ and Mock or GFP-tagged-BICD2. Nuclear and cytoplasmic extracts were isolated and analyzed by immunoblot.

Experiments were repeated at least three times reproducibly, data shown is from one repeat.

**Table S1: List of primers for qRT-PCR analysis in this study.**

| **Gene** | **Forward primer (5’->3’)** | **Reverse primer (5’->3’)** |
| --- | --- | --- |
| *Vhl* | CTCAGCCCTACCCGATCTTAC | ACATTGAGGGATGGCACAAAC |
| *Actb* | GGCTGTATTCCCCTCCATCG | CCAGTTGGTAACAATGCCATGT |
| *VHL* | GGAGCCTAGTCAAGCCTGAGA | CATCCGTTGATGTGCAATGCG |
| *HIF1a* | ATCCATGTGACCATGAGGAAATG | TCGGCTAGTTAGGGTACACTTC |
| *GAPDH* | ATGACATCAAGAAGGTGGTG | CATACCAGGAAATGAGCTTG |
| *Ifit1* | CTGAGATGTCACTTCACATGGAA | GTGCATCCCCAATGGGTTCT |
| *Ccl5* | GCTGCTTTGCCTACCTCTCC | TCGAGTGACAAACACGACTGC |
| *Cxcl10* | CCAAGTGCTGCCGTCATTTTC | GGCTCGCAGGGATGATTTCAA |
| *Isg15* | GGTGTCCGTGACTAACTCCAT | TGGAAAGGGTAAGACCGTCCT |
| *Tnf* | GAACTGGCAGAAGAGGCACT | AGGGTCTGGGCCATAGAACT |
| *Il1b* | TACCAGTTGGGGAACTCTGC | CAAAATACCTGTGGCCTTGG |
| *Il18* | GACTCTTGCGTCAACTTCAAGG | CAGGCTGTCTTTTGTCAACGA |
| *Il6* | TAGTCCTTCCTACCCCAATTTCC | TTGGTCCTTAGCCACTCCTTC |
| *Havcr1* | TCAGCTCGGGAATGCACA | TGGTTGCCTTCCGTGTCT |
| *Spp1* | AGCAAGAAACTCTTCCAAGCAA | GTGAGATTCGTCAGATTCATCCG |
| *CCL5* | CCAGCAGTCGTCTTTGTCAC | CTCTGGGTTGGCACACACTT |
| *CXCL10* | GTGGCATTCAAGGAGTACCTC | TGATGGCCTTCGATTCTGGATT |
| *OAS1* | TGTCCAAGGTGGTAAAGGGTG | CCGGCGATTTAACTGATCCTG |
| *OAS2* | ACGTGACATCCTCGATAAAACTG | GAACCCATCAAGGGACTTCTG |
| *OAS3* | TCTGAGACTCACGTTTCCTGA | CACTGTTGAGGAGGGTAGAGTA |
| *SLC3A2* | CTGGTGCCGTGGTCATAATC | GCTCAGGTAATCGAGACGCC |
| *SLC7A11* | TCTCCAAAGGAGGTTACCTGC | AGACTCCCCTCAGTAAAGTGAC |
