# Supplementary material for: Critical role of VHL/BICD2/STAT1 axis in crystal-associated kidney disease
Source: Cell Death Dis. 2023 Oct 13;14(10):680. doi: 10.1038/s41419-023-06185-1 (PMC10575931; doi:10.1038/s41419-023-06185-1)

Figure 2B

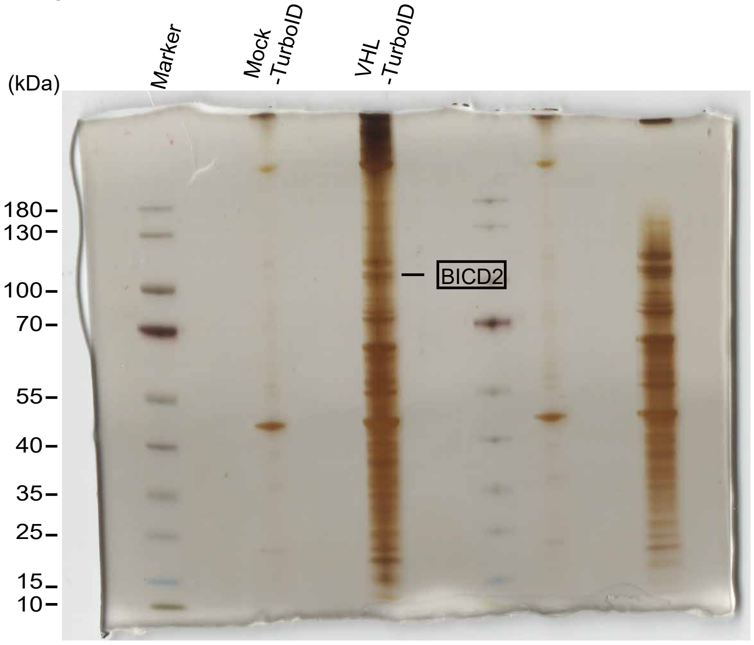

Figure 2C Left

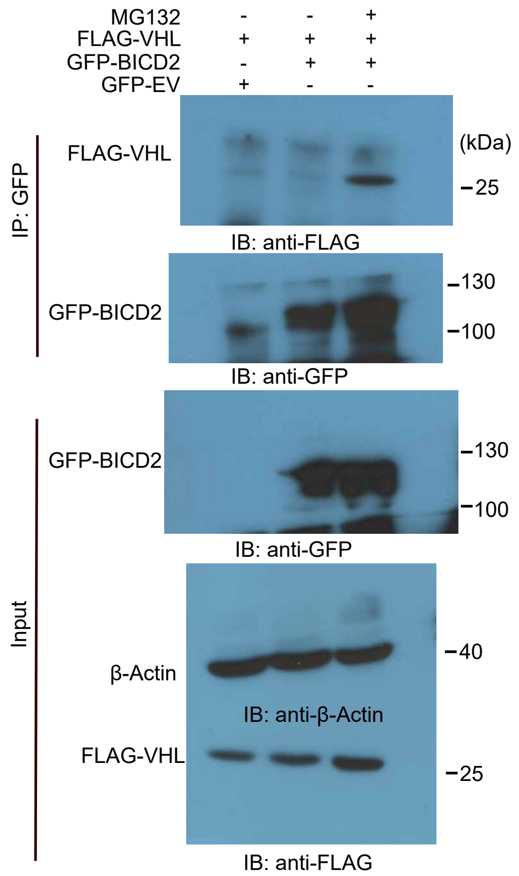

Figure 2C Right

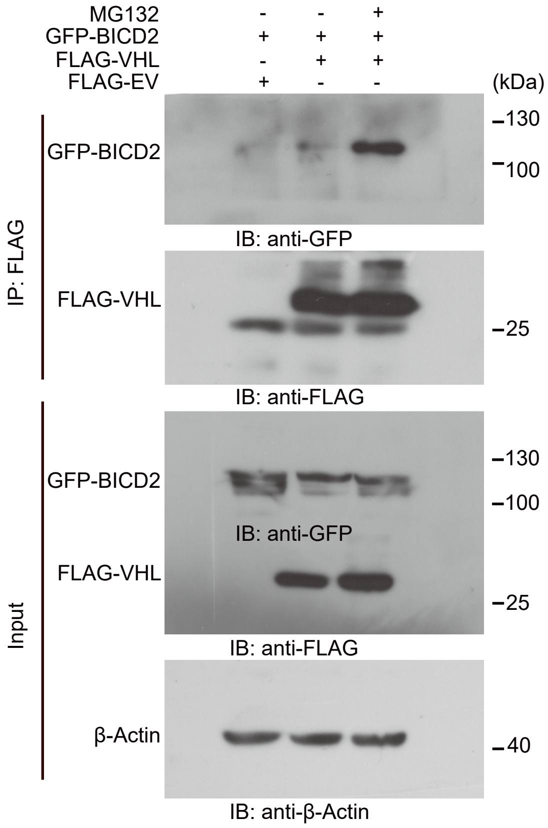

Figure 2E

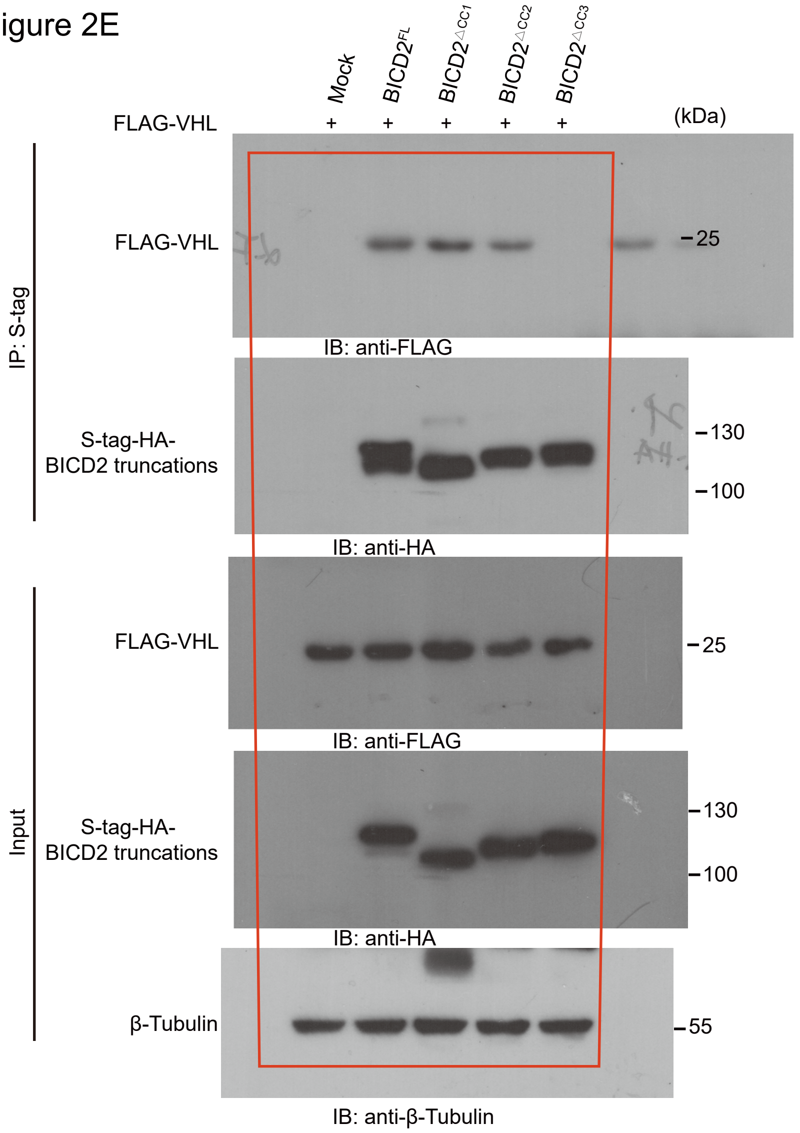

Figure 2G

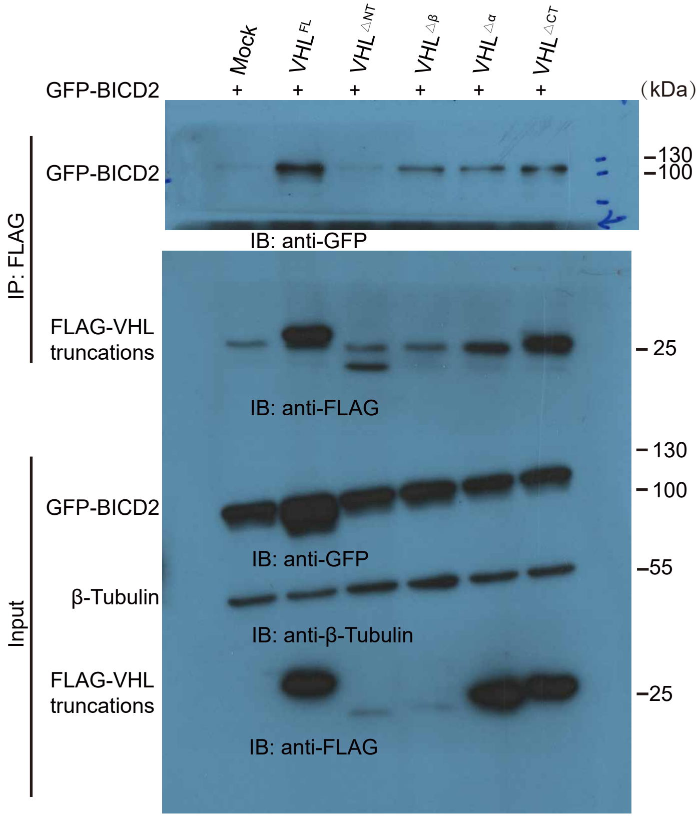

Figure 2H

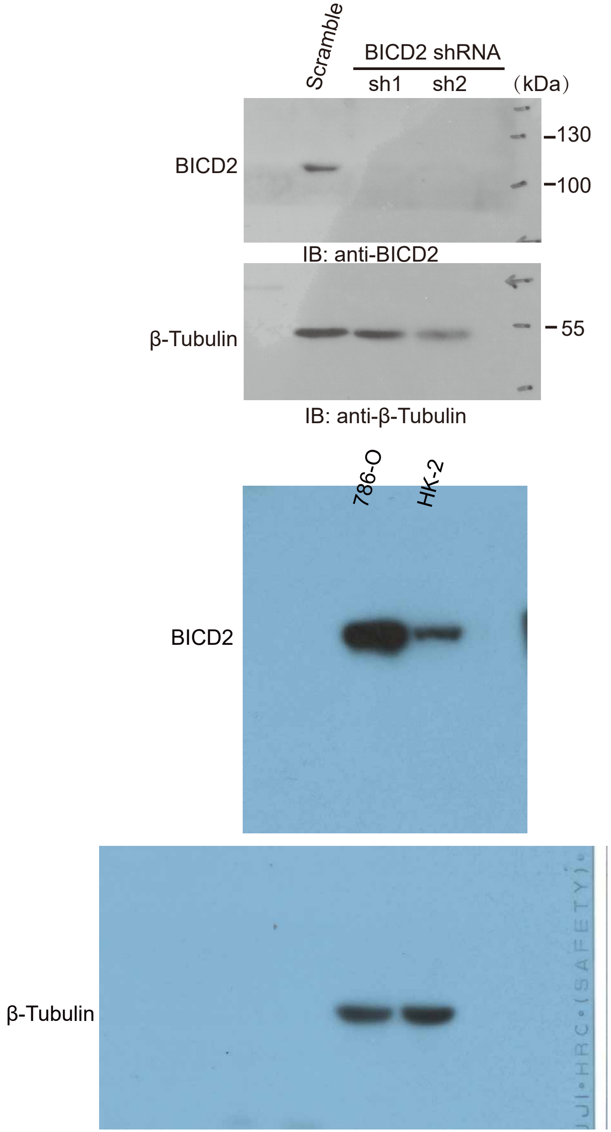

Figure 3A

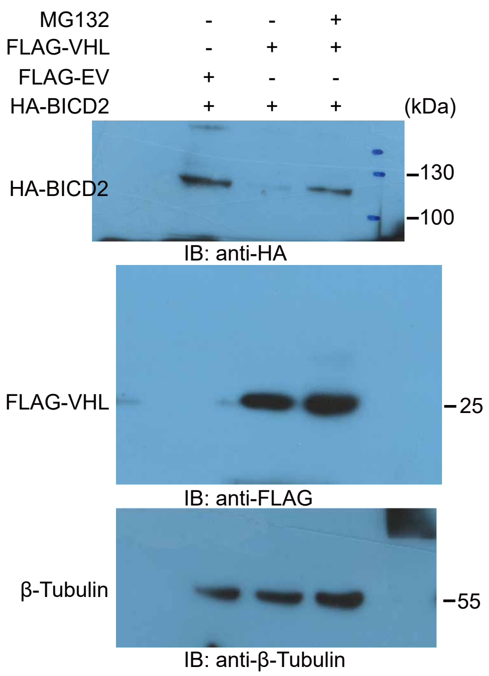

Figure 3B

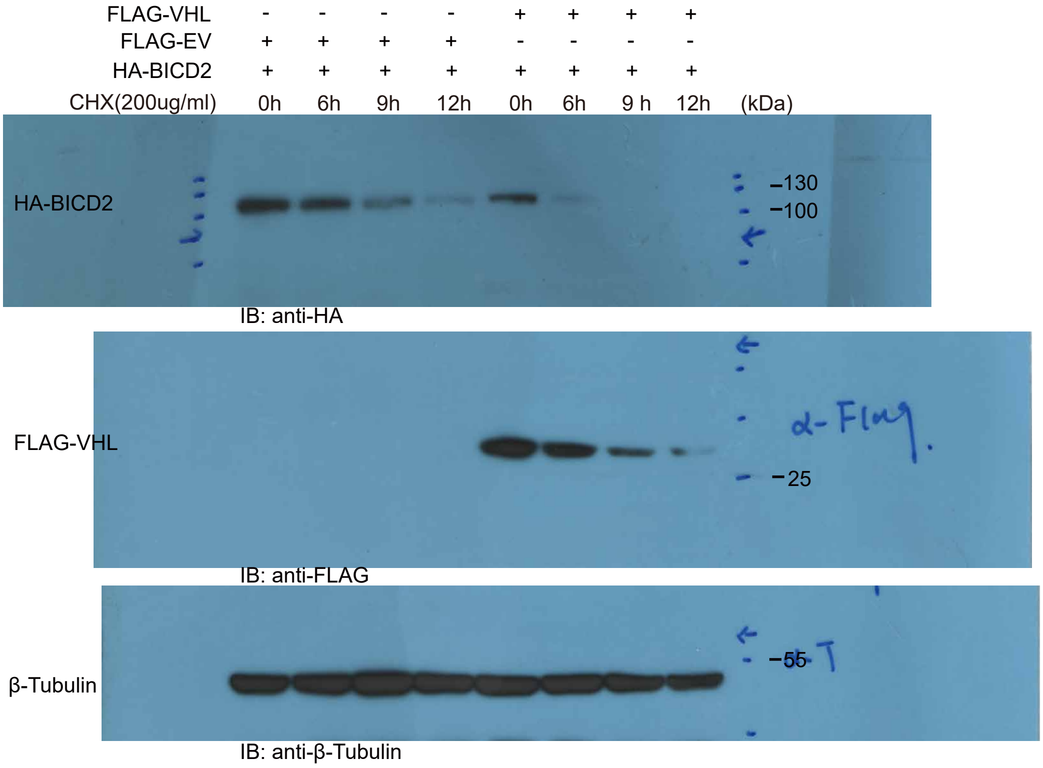

Figure 3C

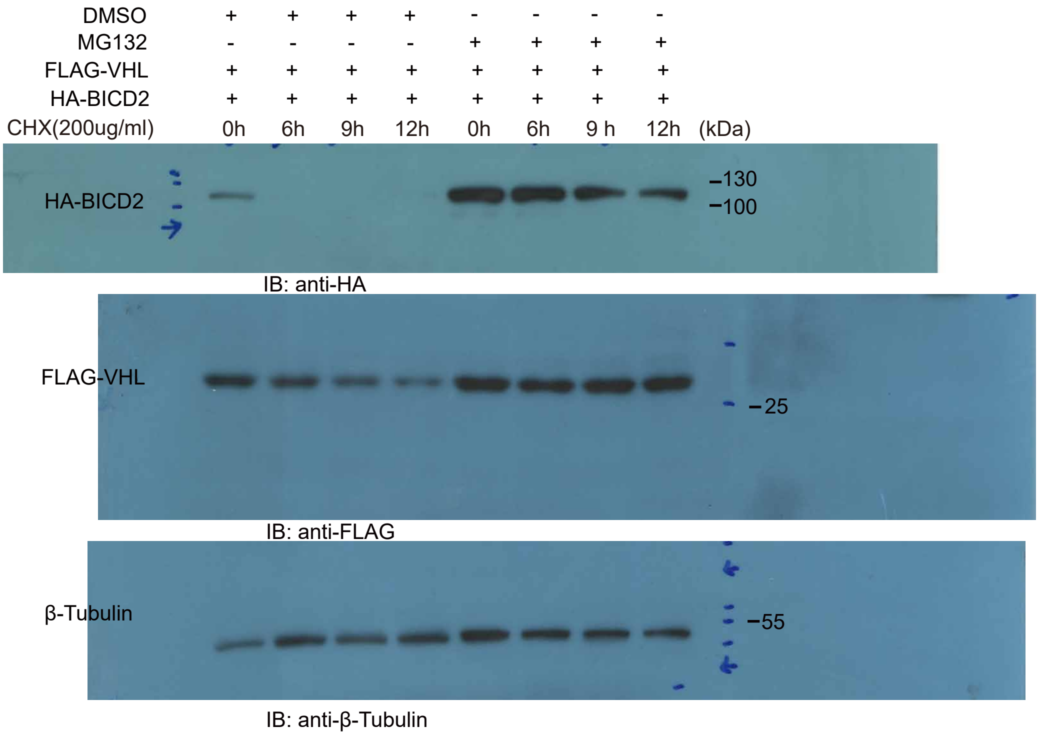

Figure 3D

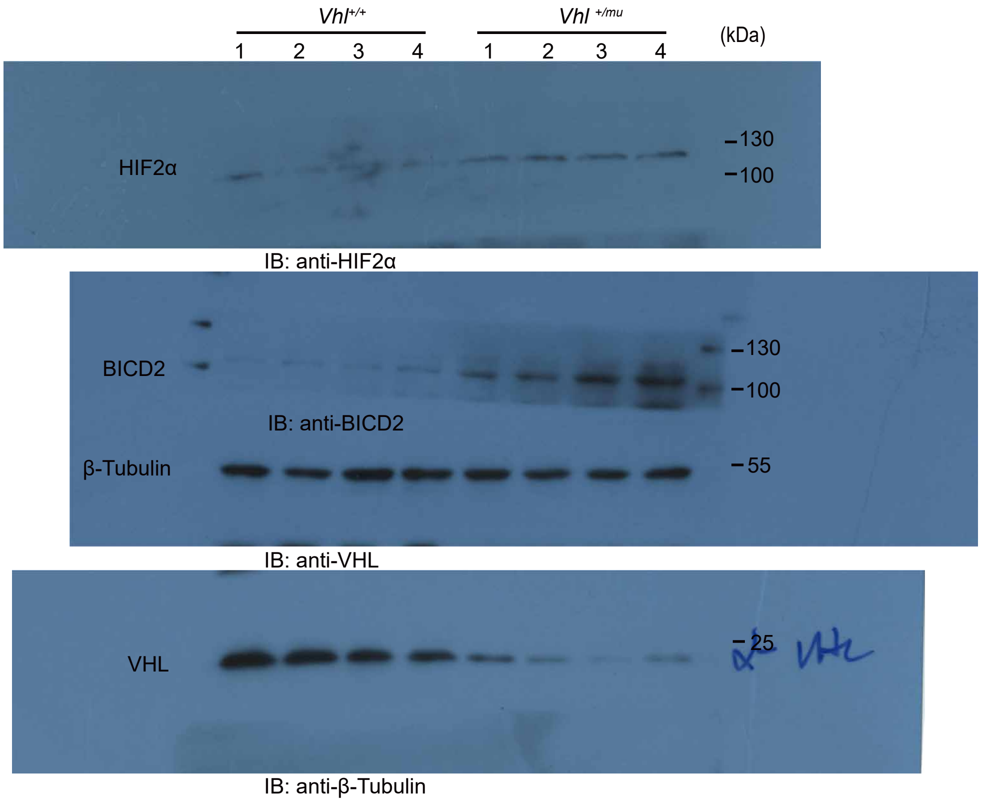

Figure 3E

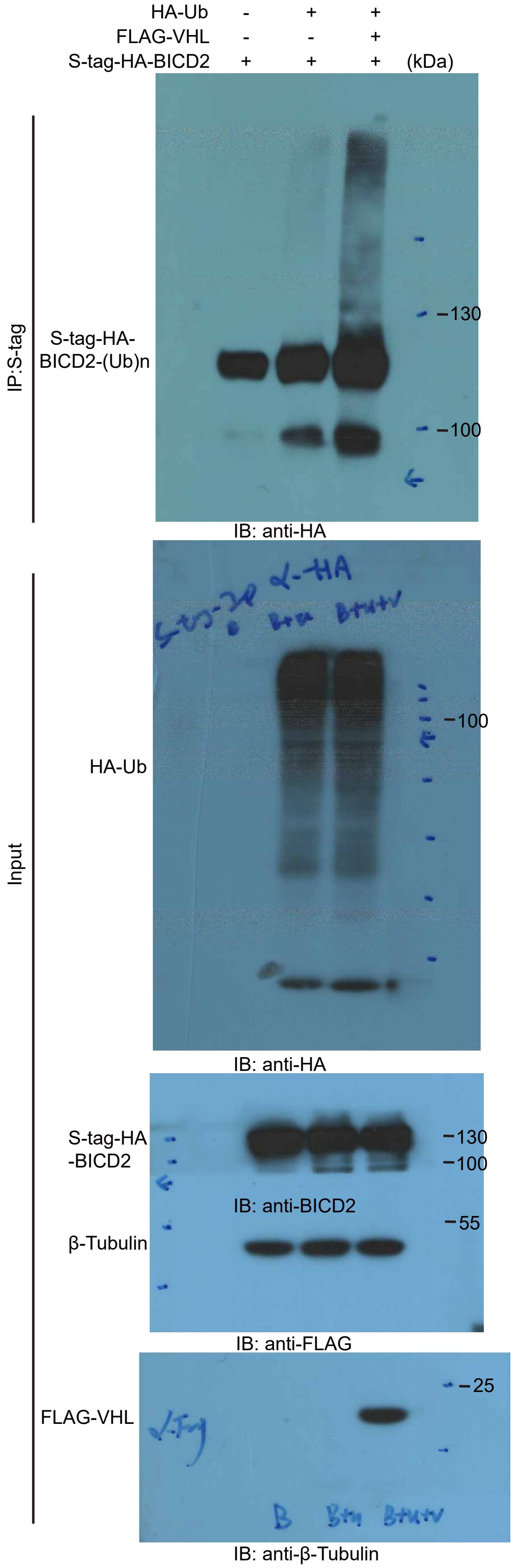

Figure 6B

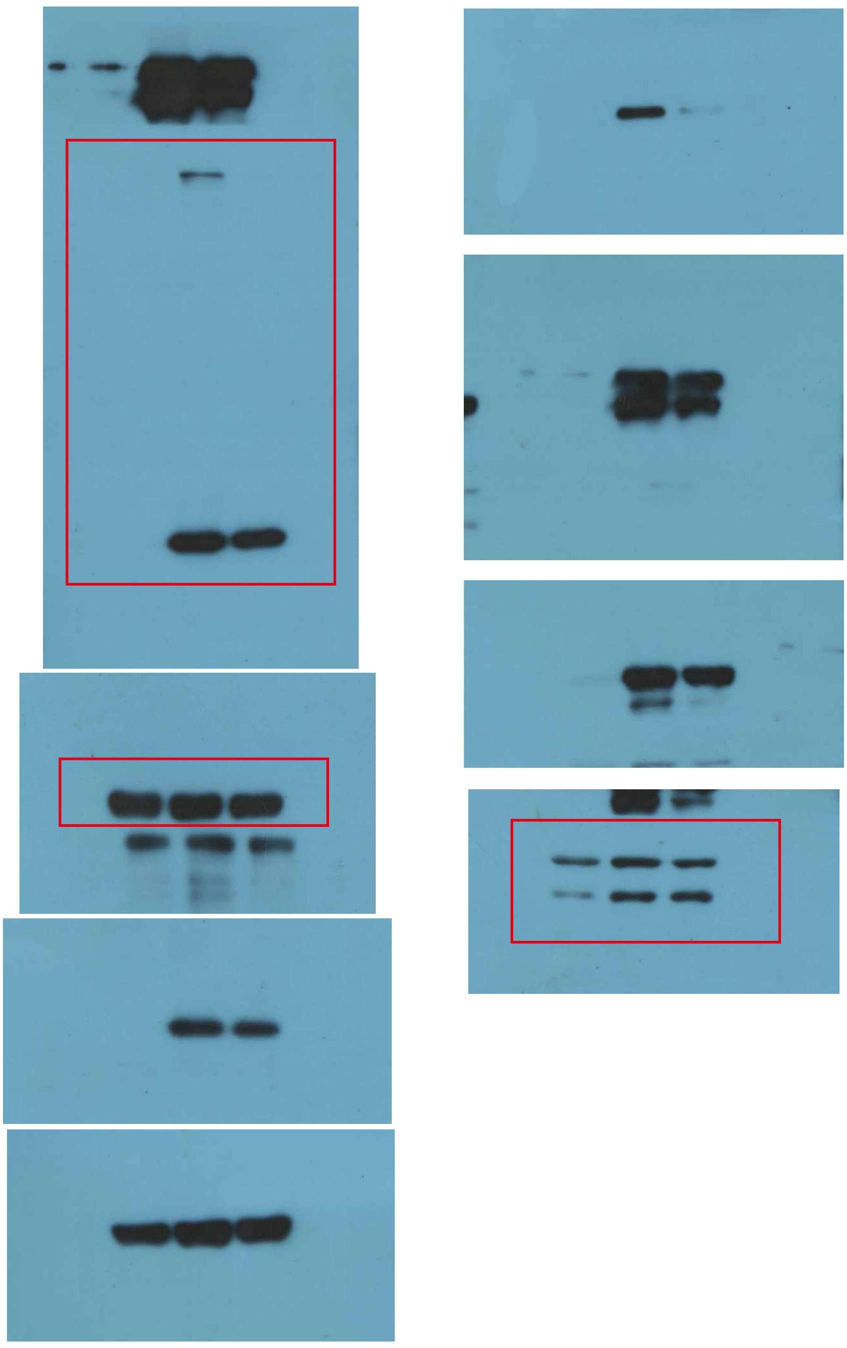

Figure 6C

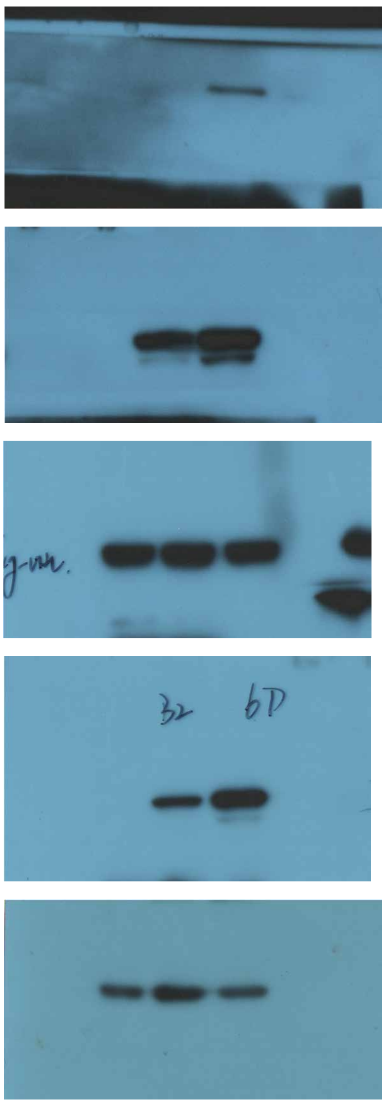

Figure 6D

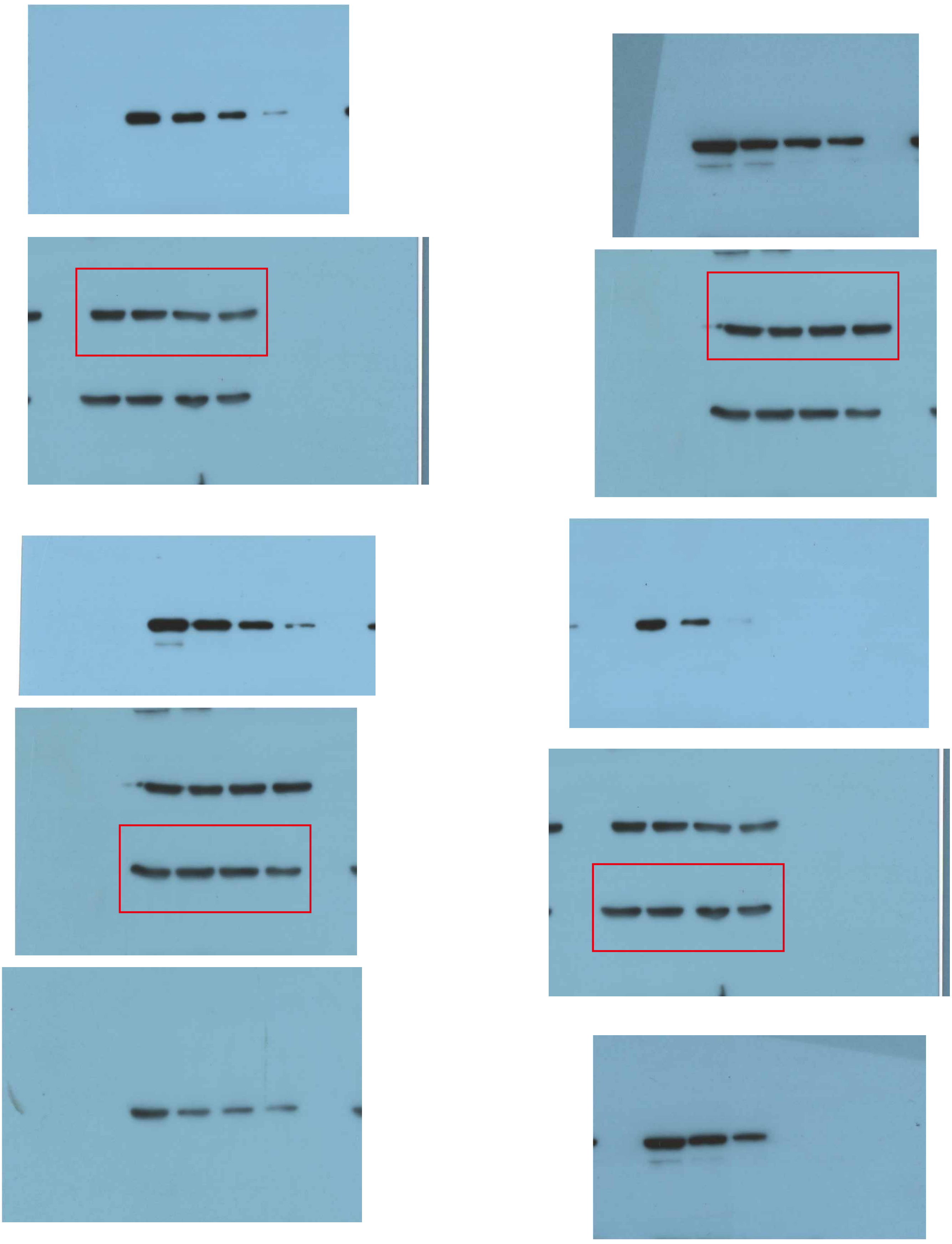

Figure 7A

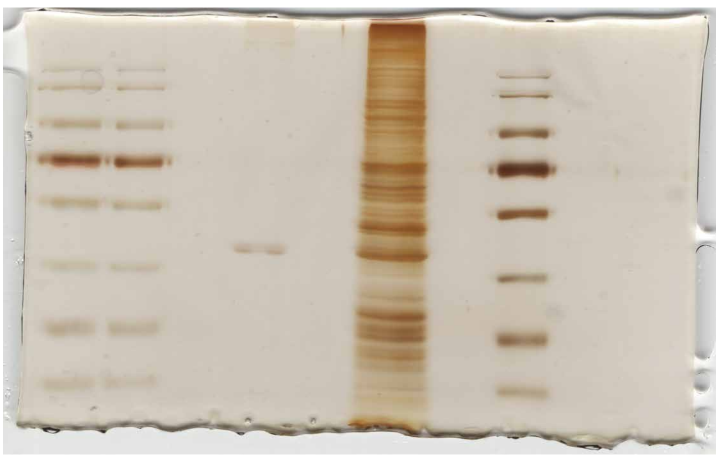

Figure 7B

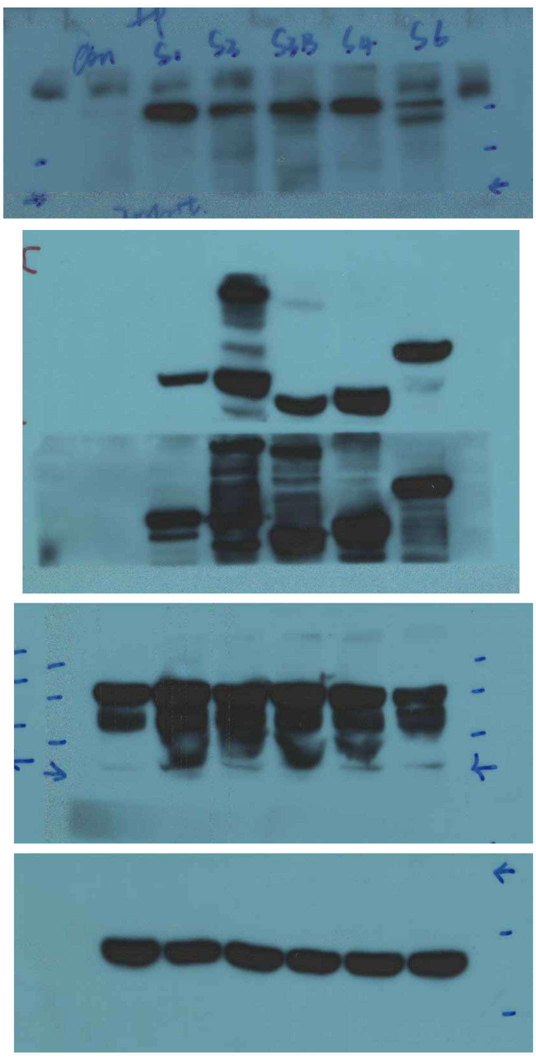

Figure 7C

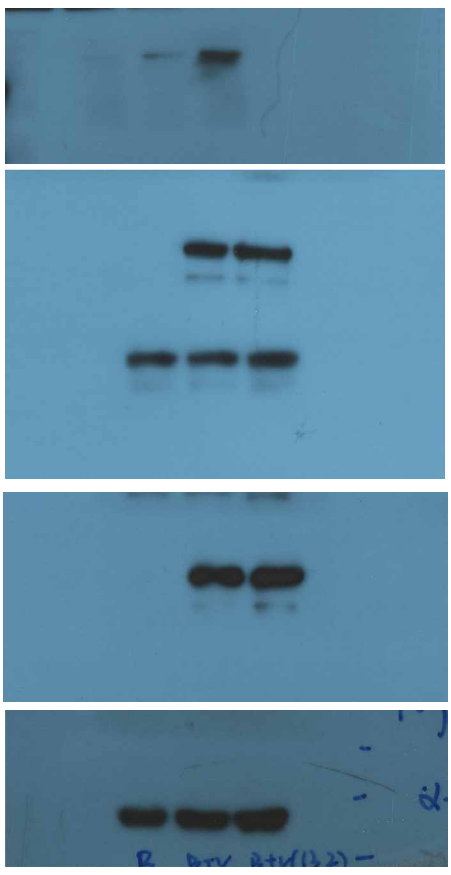

Figure 7D

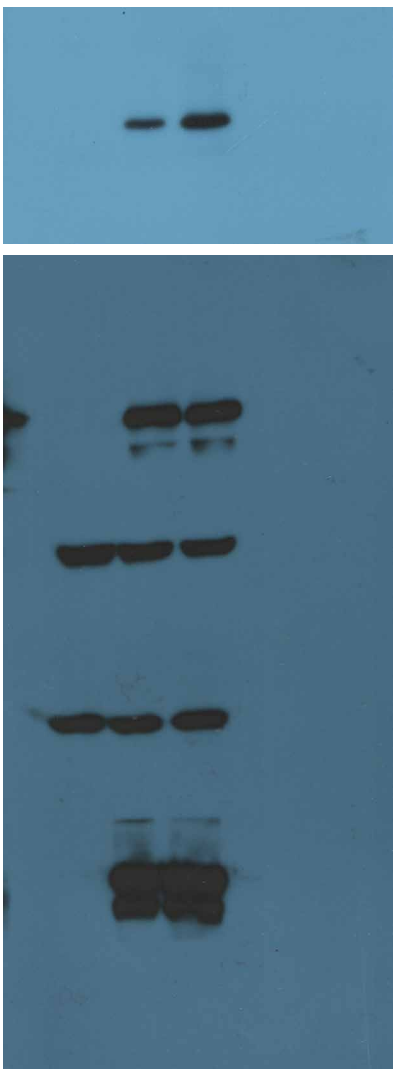

Figure 7E

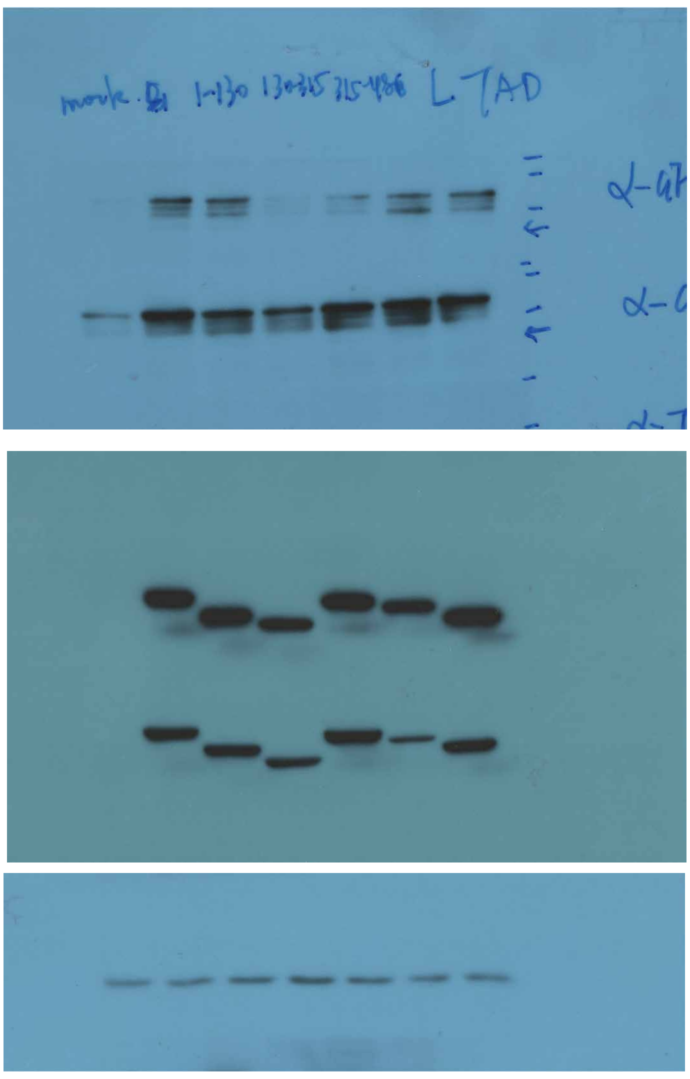

Figure 7H

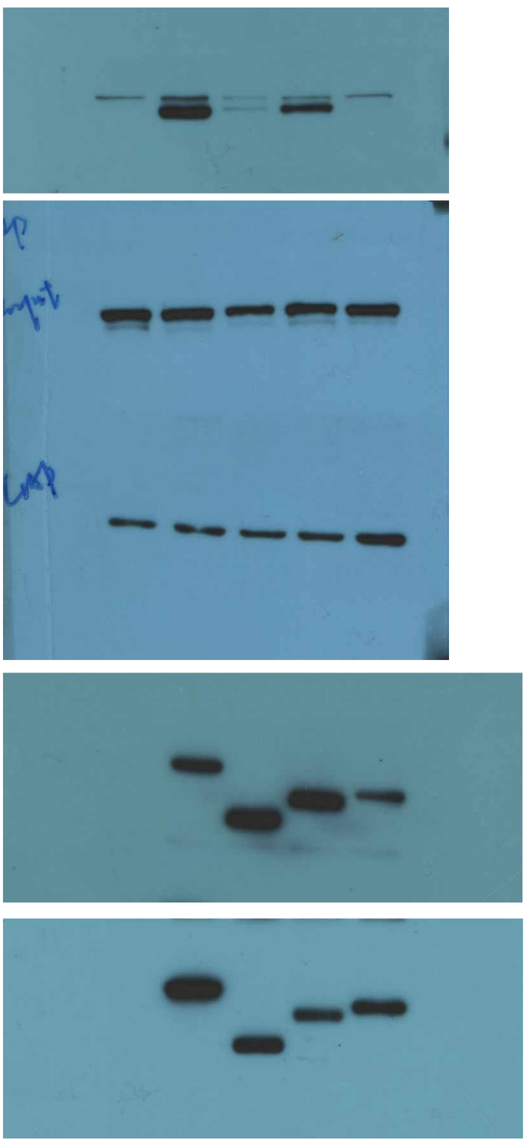

Figure 8A

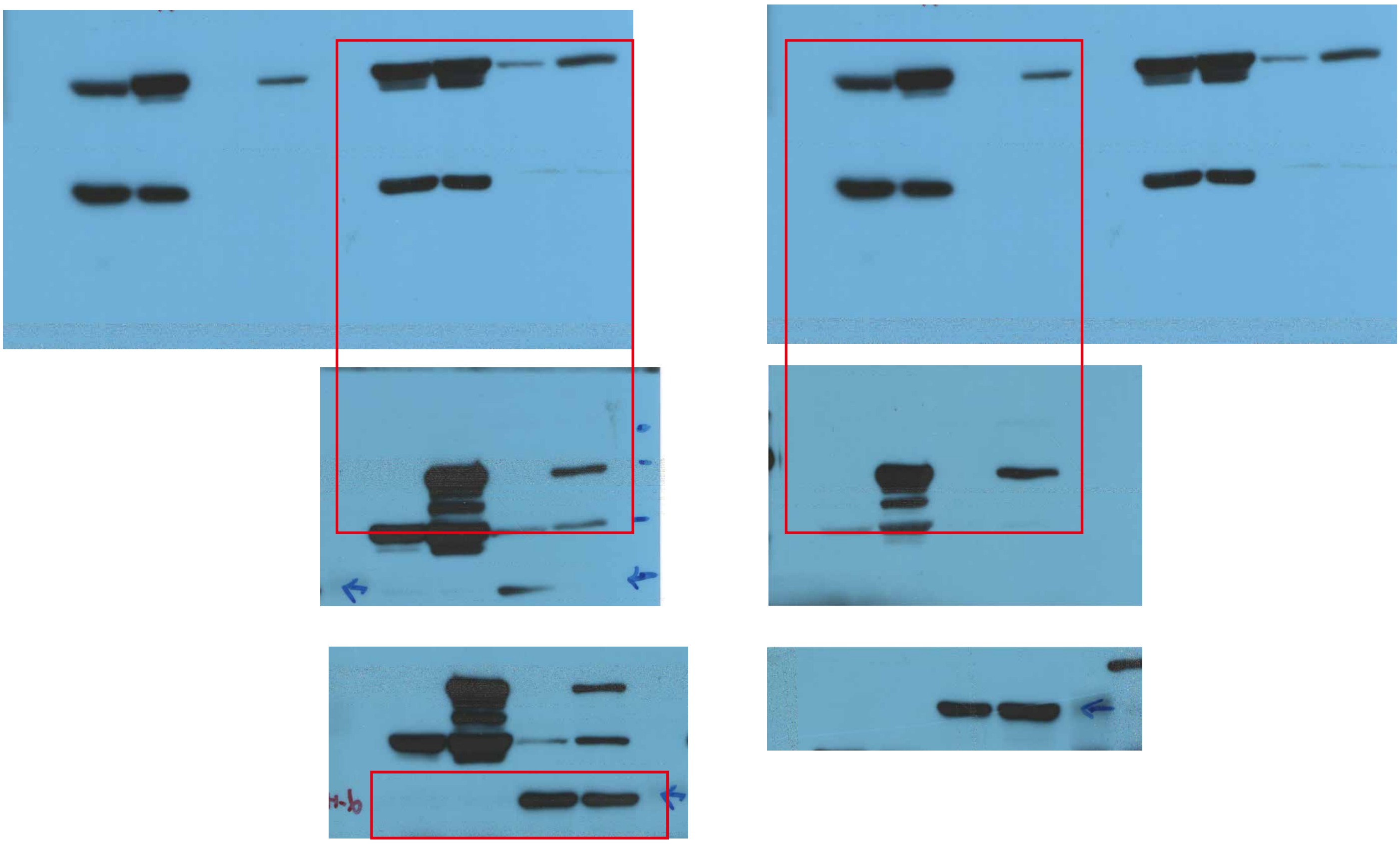

Figure S2B

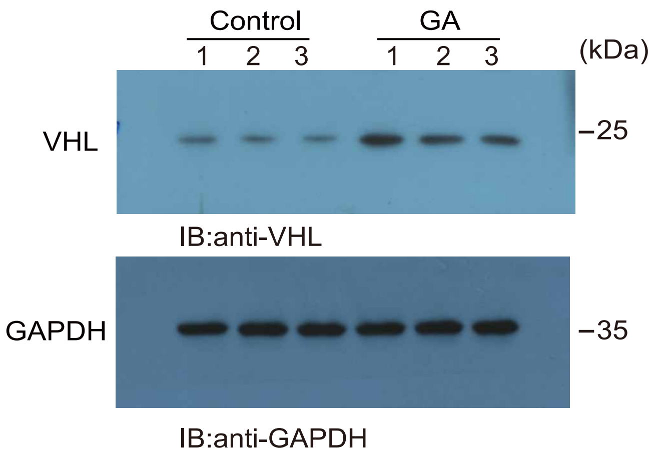

Figure S2G

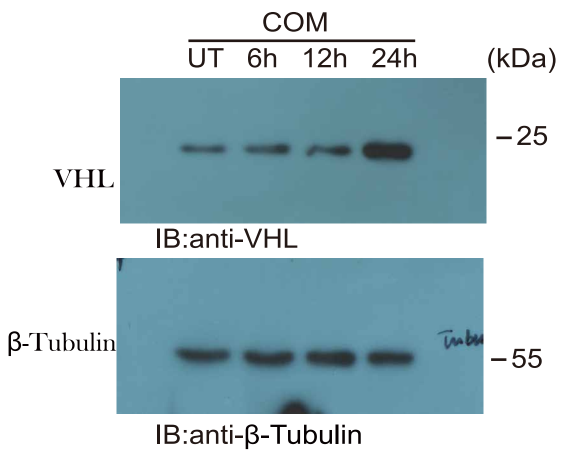

Figure S3E

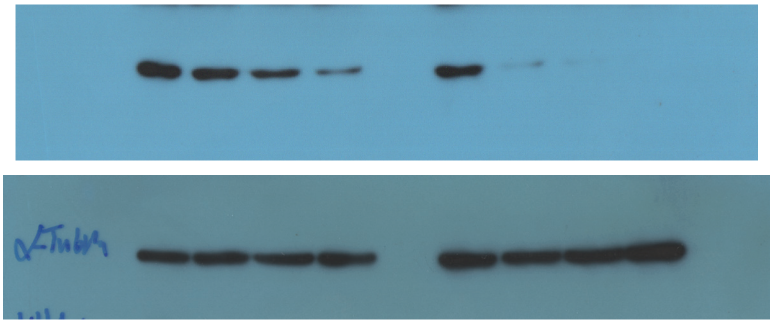

Figure S3F

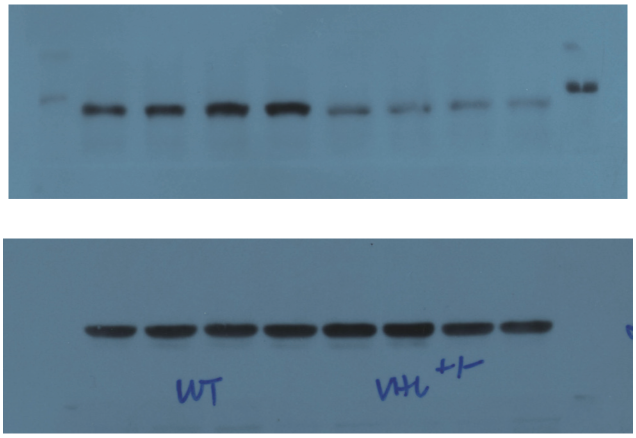

Fgiure S5D

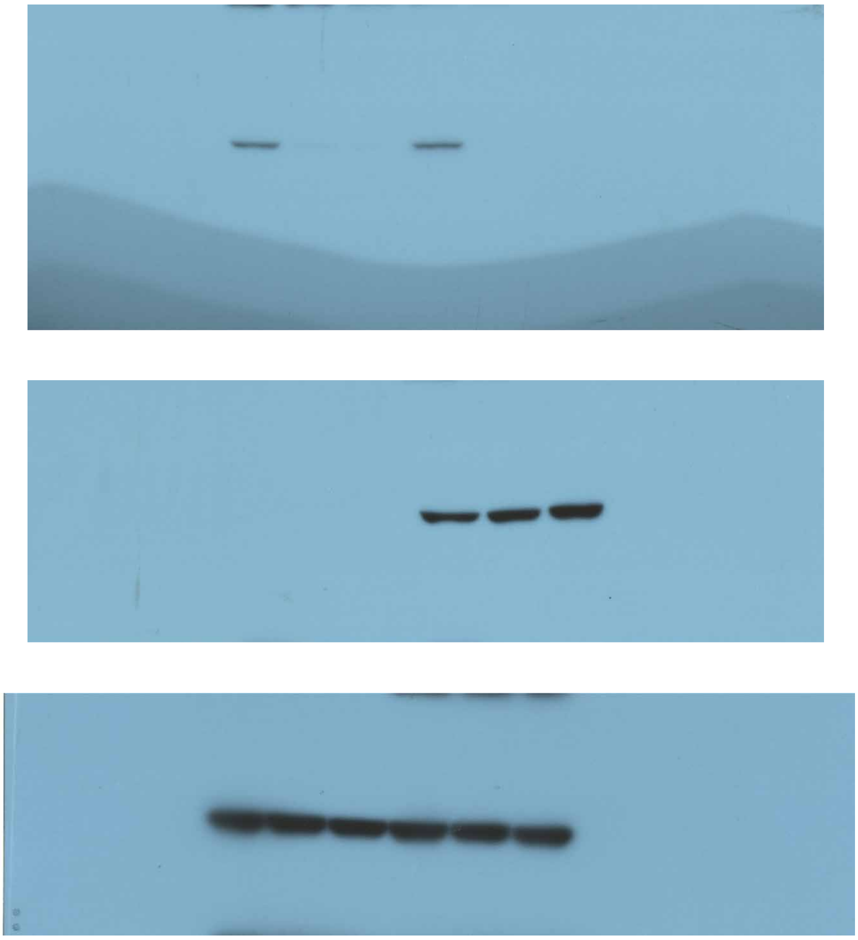

Figure S5A

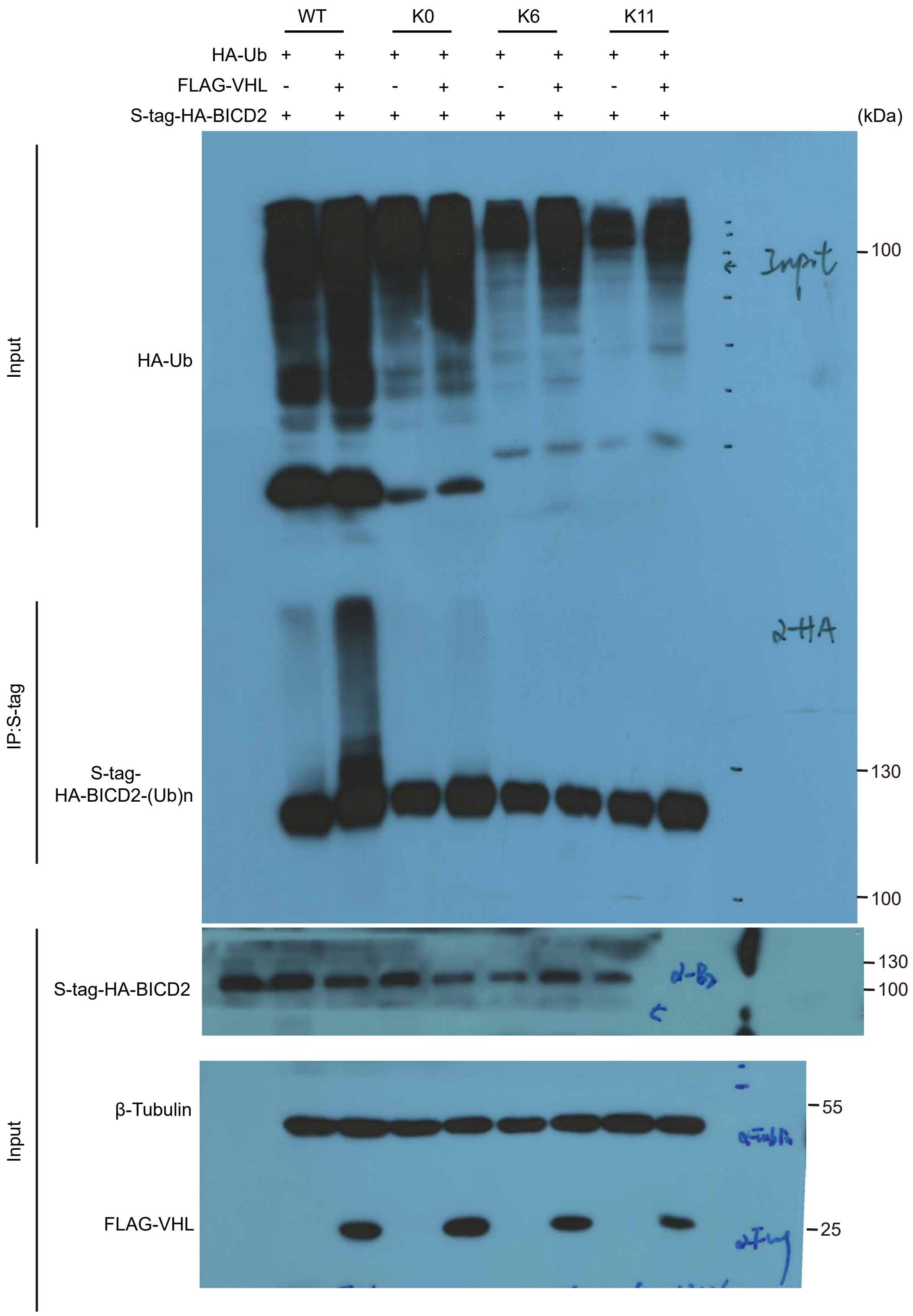

WT
K0
K6
K11
HA-Ub
FLAG-VHL
S-tag-HA-BICD2
(kDa)
Input
HA-Ub
100
IP:S-tag
S-tag-
HA-BICD2-(Ub)n
130
100
S-tag-HA-BICD2
130
100
β-Tubulin
55
FLAG-VHL
25

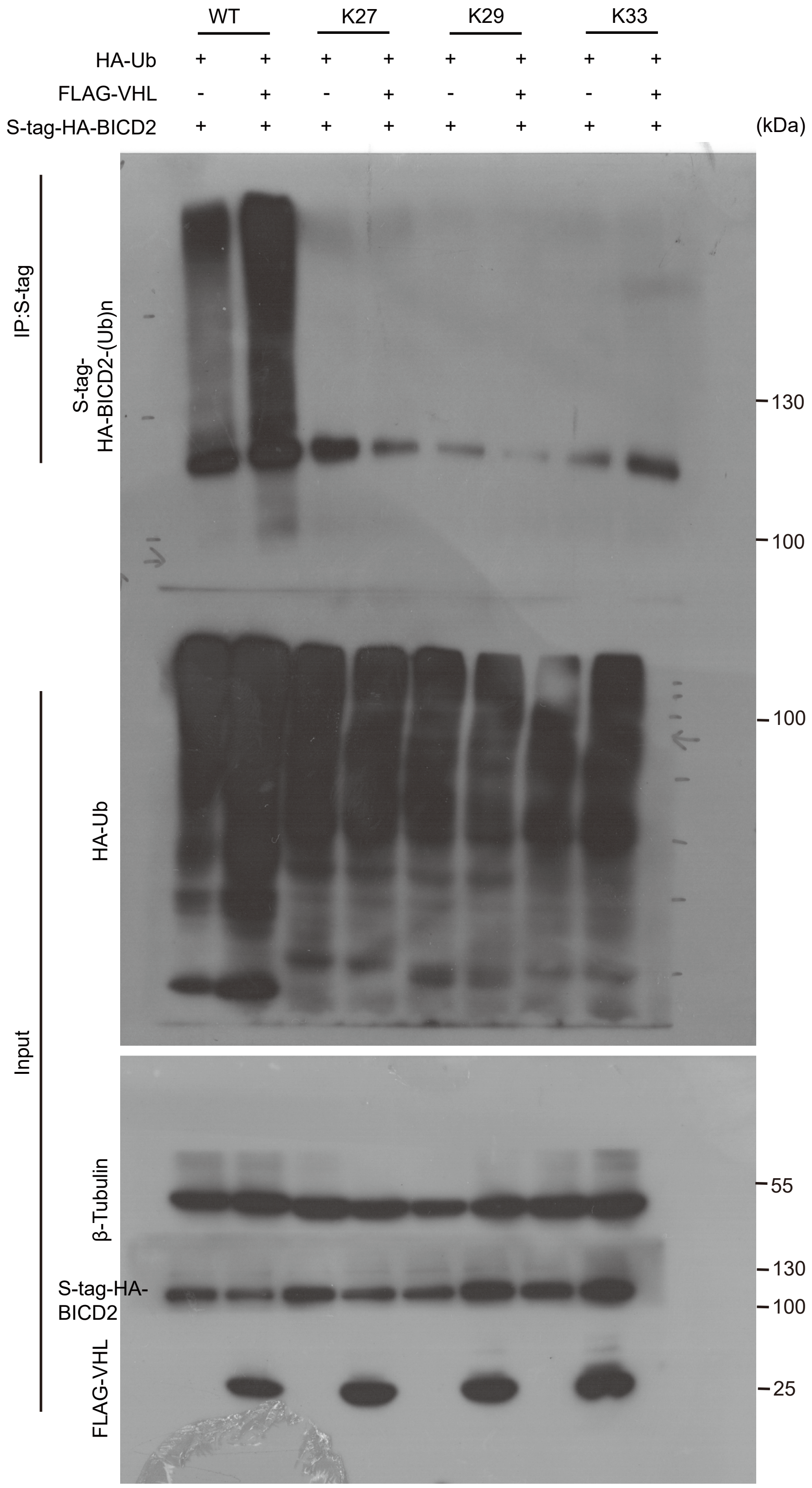
WT
K27
K29
K33
HA-Ub
FLAG-VHL
S-tag-HA-BICD2
(kDa)
IP:S-tag
S-tag-HA-BICD2-(Ub)n
130
100
Input
HA-Ub
100
β-Tubulin
55
S-tag-HA-BICD2
130
100
FLAG-VHL
25

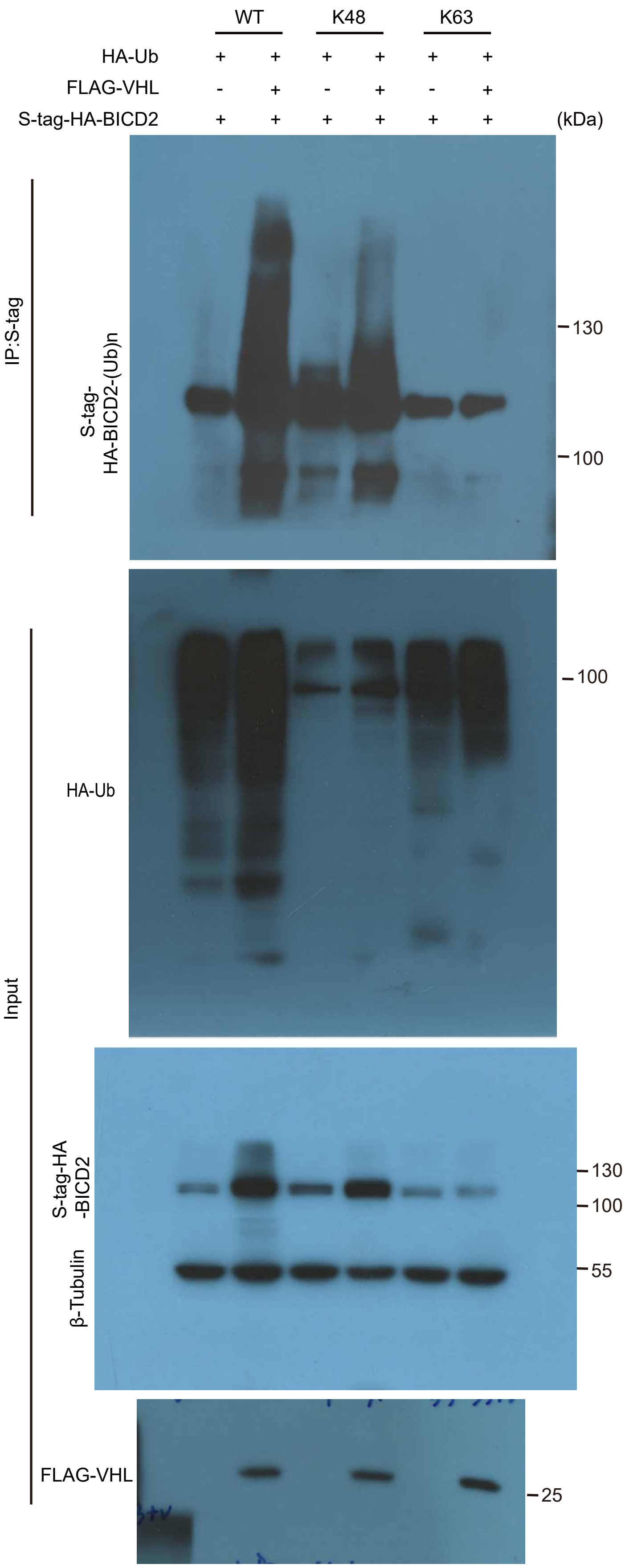
WT
K48
K63
HA-Ub
FLAG-VHL
S-tag-HA-BICD2
(kDa)
IP:S-tag
S-tag-HA-BICD2-(Ub)n
130
100
Input
HA-Ub
100
S-tag-HA-BICD2
130
100
β-Tubulin
55
FLAG-VHL
25

Figure S9A

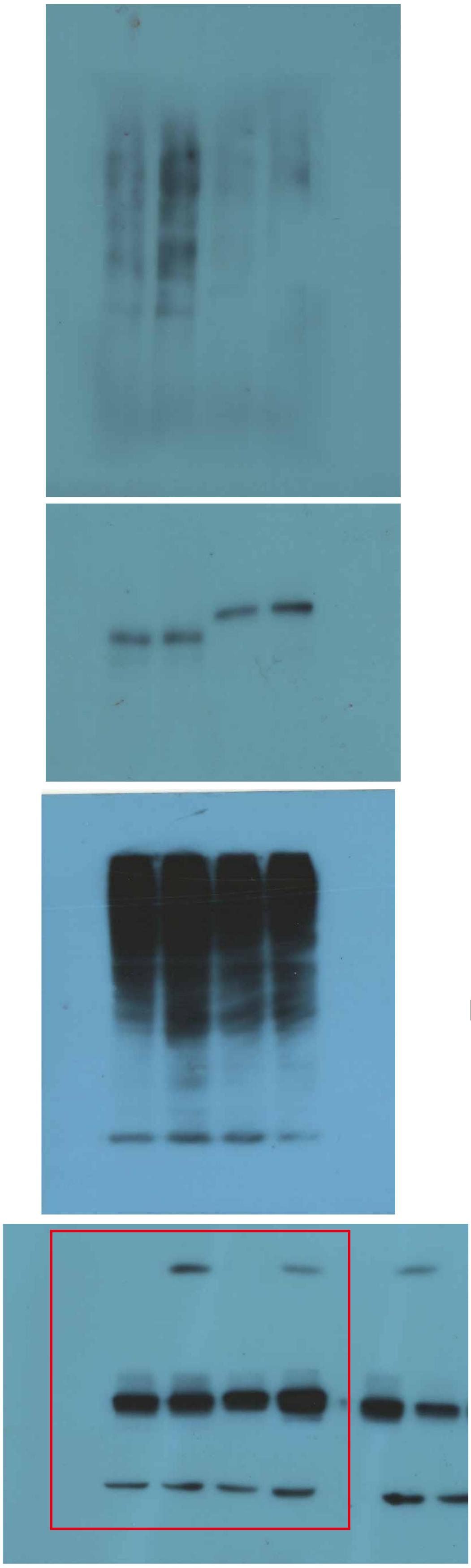

Figure S9B

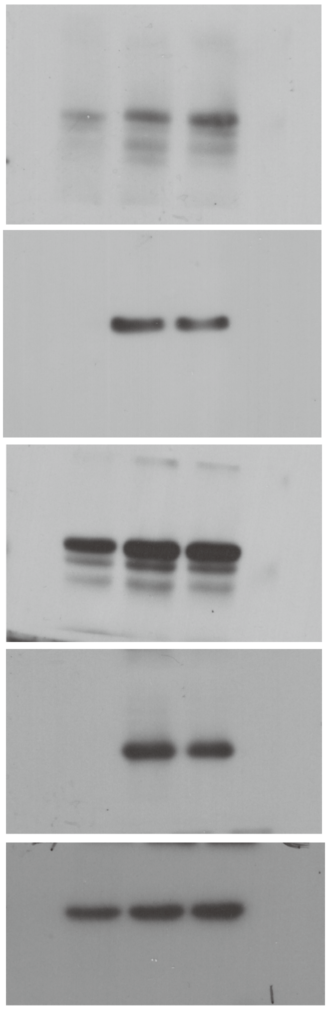

Figure S9C

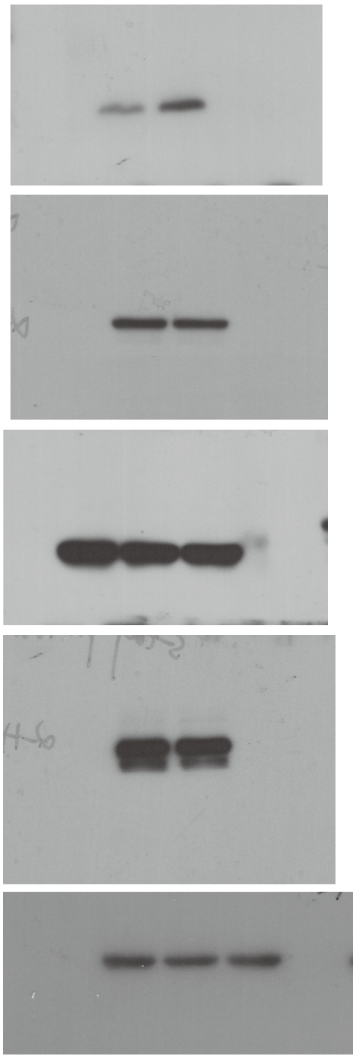

Figure S9D

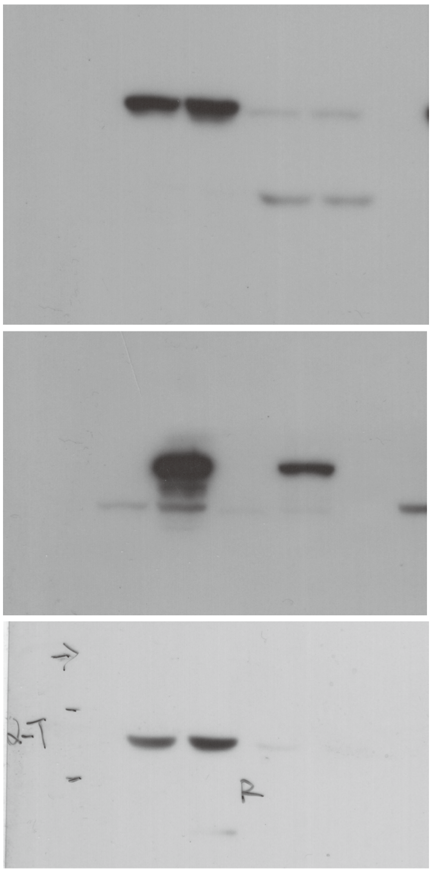

Figure S9E

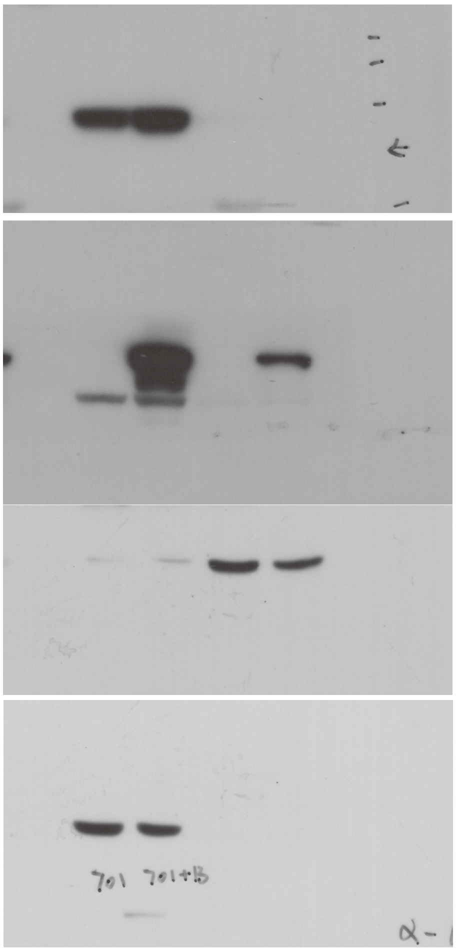

Supplement: Supplementary file 2 — Original data files [file 41419_2023_6185_MOESM2_ESM.pdf]
